# Direct coupling of lactate oxidation with butyryl-CoA formation *via* a canonical electron transfer flavoprotein in *Fusobacterium nucleatum*

**DOI:** 10.1016/j.jbc.2025.110796

**Published:** 2025-10-09

**Authors:** Long T.M. Do, Robert Godin, Kirsten R. Wolthers

**Affiliations:** Department of Chemistry, University of British Columbia, Kelowna, Canada

**Keywords:** *Fusobacterium nucleatum*, electron transfer flavoprotein, butyryl-CoA dehydrogenase, LrgAB, D-lactate dehydrogenase

## Abstract

The gram-negative opportunistic pathogen *Fusobacterium nucleatum* encodes an electron transfer flavoprotein (ETF) within a 6-gene cluster that also includes genes for a D-lactate dehydrogenase (Ldh), butyryl-CoA dehydrogenase (Bcd), and LrgAB. Herein, we demonstrate that ETF functions as a canonical ETF, transferring two electrons from Ldh following oxidation of D-lactate to Bcd for the reduction of crotonyl-CoA to butyryl-CoA. Steady-state kinetic analysis of the Ldh_FN_/ETF_FN_/Bcd_FN_ reaction (lactate + crotonyl-CoA → pyruvate + butyryl-CoA) yielded a *k*_cat_ of 2.5 ± 0.1 s^−1^ and a *K*_M_ of 0.65 ± 0.04 μM and 5.2 ± 0.5 μM for D-lactate and butyryl-CoA, respectively. As observed in homologous ETFs, the flavin adenine dinucleotide (FAD) cofactor of ETF forms the red anionic semiquinone (FAD^•-^) but the *E*^o^′ values (*versus* the normal hydrogen electrode) of −70 mV (FAD/FAD^•-^) and = −122 mV (FAD^•-^/FADH^-^) are more compressed and negative compared to other ETFs, indicating the flavoprotein is physiologically primed to accept two electrons from Ldh. Similarly, reductive titration of Ldh shows that its FAD cofactor also forms the red anionic semiquinone, but the *E*^o^′ values for FAD/FAD^•-^ (−109 mV) and FAD^•-^/FADH^-^ (−115 mV) are even more closely spaced. We discuss how *F. nucleatum* potentially uses this lactate utilization gene cluster to maintain redox homeostasis during oxidative stress and how beneficial gut anaerobes of the Lachnospiraceae family with similar gene clusters employ either a canonical or bifurcating ETF for the conversion of lactate (and acetate) to butyrate.

Electron transfer flavoproteins (ETFs)—widely distributed throughout the kingdom of life)—function as electrical conduits for diverse biological processes. ETFs are comprised of an α- and β-subunit that combine to form a three-domain heterodimer (1, 2, 3, 4). The N- and C-terminal halves of the larger α-subunit form domains I and II, respectively, while the small β-subunit forms domain III. ETFs are divided into two classes: canonical and bifurcating. The former class is well known to function in mitochondrial respiration by transferring electrons from nine different flavin adenine dinucleotide (FAD)-containing acyl-CoA dehydrogenases of fatty acid β-oxidation and amino acid catabolism to the ubiquinone pool of the main respiratory chain *via* the integral membrane protein, ETF-ubiquinone oxidoreductase (5, 6, 7, 8, 9, 10). Canonical ETFs can also accept reducing equivalents from sarcosine dehydrogenase and dimethylglycine dehydrogenase, two enzymes of mitochondrial one-carbon metabolism (11), and from trimethylamine dehydrogenase in *Methylophilus methylotrophus* (12). Canonical ETFs contain an AMP group bound to the β-subunit and FAD cofactor in the α-subunit; the latter cofactor mediates electron transfer between redox partner proteins (13, 14).

Bifurcating ETFs also contain an FAD in the α-subunit (designated α-FAD), but an FAD replaces the AMP in the β-subunit (15, 16). This second FAD in the β-subunit (designated β-FAD) underpins the function of bifurcating ETFs in energy conservation, as it facilitates the coupling of an endergonic redox reaction with an exergonic one (17). The first descriptions of an ETF functioning in flavin-based electron bifurcation were from the obligate anaerobes *Acidaminococcus fermentans* and *Clostridium kluyveri* (18, 19, 20). The ETFs from these organisms form a complex with a butyryl-CoA dehydrogenase (Bcd–ETF), where they couple the endergonic NADH-dependent (−320 mV) reduction of ferredoxin/flavodoxin (−500 mV) with NADH-dependent reduction of crotonyl-CoA to butyryl-CoA (−10 mV) (21). The overall reaction initiates with the β-FAD accepting a hydride from NADH, leading to formation of a two-electron–reduced hydroquinone (β-FADH^-^) and NAD^+^ (22). β-FADH^-^ subsequently splits (or bifurcates) the electrons with one electron reducing the red anionic semiquinone form of ⍺-FAD (⍺-FAD•^-^) to the anionic hydroquinone (⍺-FADH^-^) and the second electron reducing ferredoxin or flavodoxin. The ⍺-FADH^-^ then transfers its electron further to the FAD cofactor of Bcd (termed *δ*-FAD). After a second round of NADH oxidation and electron bifurcation, the completely reduced *δ*-FADH^-^ transfers a hydride to crotonyl-CoA to yield butyryl-CoA. Homologs of Bcd-ETF are widespread in obligate anaerobic bacteria, where they function to generate reduced ferredoxin/flavodoxin, which is used for energy conservation *via* the Na^+^-pumping ferredoxin-NAD^+^ reductase, also known as Rnf (23, 24).

The gram-negative anaerobe, *Fusobacterium nucleatum* subsp. *polymorphum* American tissue culture collection (ATCC) 10953 encodes two sets of ETFs (FN0784-FN0785 and FN1533-FN1534), which are present in distinct gene clusters (Fig. 1). FN0784-FN0785 form a tricistron with the third gene (FN0783) encoding a Bcd. The second ETF (hereafter labeled ETF_FN_) is part of a 6-gene cluster (FN1531-1536), which also includes genes that encode LrgA (FN1532), LrgB (FN1531), lactate dehydrogenase (Ldh_FN_; FN1536), and a Bcd (Bcd_FN_; FN1535). We have termed this gene cluster *lct* (lactate utilization) because it resembles the *lct* gene cluster described for *Anaerostipes hadrus*, *Anaerostipes caccae*, *Anaerobutyricum hallii*, *Coprococcus catus, and Clostridium beijerinckii,* a small subset of Firmicutes that convert lactate and acetate to butyrate (25, 26). A similar gene cluster is found in *Fusobacterium necrophorum* subsp. *necrophorum*, which is associated with liver abscesses and foot rot in animals (27). Studies of *lrgAB* in *Staphylococcus aureus*, *Bacillus* spp, and *Streptococcus mutans* reveal that the two gene products are involved in the transport of pyruvate, and that the expression of the proteins is controlled by a LytSR two-component signal transduction system (28, 29, 30, 31, 32, 33, 34, 35, 36). In *S*. *mutans*, O_2_ exposure leads to an increase in *lrgAB* expression, and a Δ*lrgAB* mutant is more susceptible to oxidative stress, suggesting that the transport of pyruvate by LrgAB is part of the oxidative stress response (35, 37). Notably, oxidative stress also appears to influence the expression of *lrgAB* in *F. nucleatum*. Studies by Scheible *et al.* revealed that ModRS (homologous to LytSR), a two-component signal transduction system that functions as a global regulator of the oxidative stress response, also controls the expression of all six contiguous genes at the FN1531-1536 gene locus (Fig. 1) (38).

**Figure 1:**
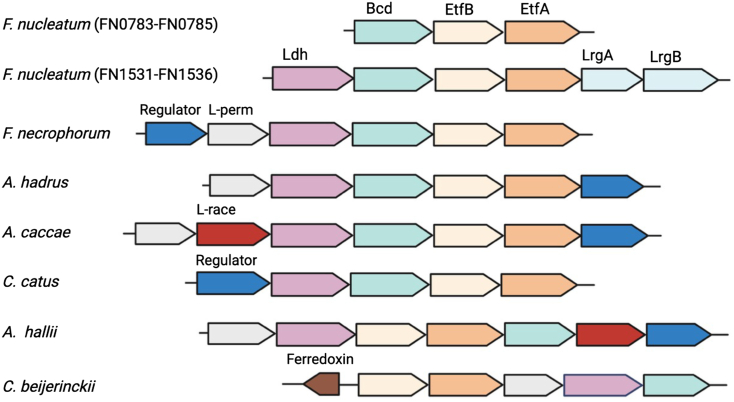
**Genetic organization of two sets of EtfAB genes in *Fusobacterium nucleatum* and the organization of lactate utilization loci observed in *F. nucleatum* and other lactate-utilizing bacteria.** Butyryl-CoA dehydrogenase (Bcd), β- and α-subunits of electron transfer flavoprotein (EtfB and EftA), NAD^+^-independent D-lactate dehydrogenase (Ldh), lactate operon transcriptional regulator (regulator), lactate permease (L-perm), and lactate racemase (L-race).

Given that the gene locus FN1531-1536 appears to be part of an oxidative stress defence response, we were interested in the role of the ETF_FN_ encoded at this particular locus. Based on its genetic linkage to Ldh_FN_ and Bcd_FN_, we hypothesize that ETF_FN_ may function as an electron conduit between the two dehydrogenases. Both canonical and bifurcating ETFs are known to interact with homologs of Bdh_FN_ and Ldh_FN_, accepting or donating single electrons *via* the ⍺-FAD cofactor (21, 39, 40). Given that the midpoint reduction potential for lactate/pyruvate (−190 mV) is more negative than that of butyryl-CoA/crotonyl-CoA (−10 mV), we hypothesize that ETF_FN_ is used to couple lactate oxidation to crotonyl-CoA reduction. Our results confirm that ETF_FN_ functions as a canonical ETF, transferring two electrons from reduced Ldh_FN_, generated following the oxidation of D-lactate, to oxidized Bcd_FN,_ for the reduction of crotonyl-CoA to butyryl-CoA. Critically, these results uncover a novel mechanism by which *F. nucleatum* can maintain redox balance during oxidative stress.

## Results

### Characterization, purification, and optical properties of ETF_FN_

A sequence alignment of the ETF β-subunit corresponding to FN1533 and FN0784 with canonical ETFs from *Homo sapiens* (2), *M*. *methylotrophus* (41), and *Paracoccus denitrificans* (3), and bifurcating ETFs from *Clostridium difficile* (21)*, A*. *fermentans* (19), and *Acetobacterium woodii* (42) reveals that FN1533 contains a phenylalanine (Phe130) in place of a conserved glycine (Gly123 using *A. fermentans* numbering) that is invariant in bifurcating ETF (Fig. S1). Structural studies have shown that a small side chain at this position minimizes steric conflict with the isoalloxazine ring of β-FAD (43, 44). Canonical ETFs tend to have bulkier residues at this position, which thwarts the binding of the flavin mononucleotide portion of β-FAD. Thus, based on this alignment, ETF encoded by FN1533-FN1534 binds an AMP in the β-subunit and therefore likely functions as a canonical ETF. In contrast, the β-subunit of FN0784 encodes a glycine (Gly123) at the corresponding position, and thus is expected to participate in flavin-based electron bifurcation, as described for the Bcd–ETF complexes of *A. fermentans* and *C. kluyveri* (18, 19, 20).

**Figure 2:**
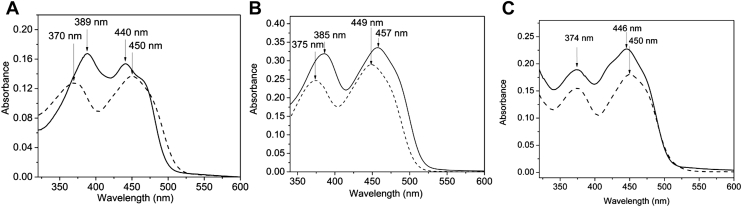
**Optical properties of the three flavoproteins.***A*, UV-absorbance spectra of ETF_FN_ (*black line*) and of free FAD following heat denaturation of ETF_FN_ (*dotted line*). *B*, UV-absorbance spectra of Ldh_FN_ (*black line*) and of free FAD following heat denaturation of Ldh_FN_ (*dotted line*). *C*, UV-absorbance spectra of Bcd_FN_ (*black line*) and of free FAD following heat denaturation of Bcd_FN_ (*dotted line*). Bcd, butyryl-CoA dehydrogenase; ETF, electron transfer flavoprotein; Ldh, lactate dehydrogenase.

Recombinant expression of ETF_FN_ (FN1533-FN1534) in *Escherichia coli* BL21(DE3) followed by purification using Ni-nitrilotriacetic acid (Ni-NTA) chromatography and anion exchange chromatography yielded a protein with an electronic absorption spectrum diagnostic of a flavoprotein with ƛ_max_ at 389 and 440 nm, corresponding to bands II and I, respectively (Fig. 2*A*). As observed in other canonical ETF preparations, the amplitude of band II is higher than that of band I and band II is more red-shifted in comparison to other flavoproteins, resulting in a shorter and shallower separation between the two absorption bands (19, 45, 46). The released flavin produced following heat denaturation of the protein exhibited ƛ_max_ values at 370 and 450 nm, diagnostic of the FAD cofactor. The ratio of FAD to protein was calculated to be 0.5:1, and the calculated extinction coefficient of oxidized ⍺-FAD bound to ETF is 13,721 M^−1^ cm^−1^. A 10% denaturing polyacrylamide gel showed two proteins with approximate molecular masses of 38 kDa (EtfA) and 34 kDa (EtfB), which are modestly higher than the calculated mass of EtfA (34,980 Da) and EtfB with the N-terminal hexahistidine tag (30,560 Da) (Fig. S2). Aberrant migration of both subunits may be attributed to their acidic properties (pI < 5.2). Approximately 50 mg of homogenous ETF was obtained from 1 L of bacterial culture.

### Characterization, purification, and optical properties of Ldh_FN_ and Bcd_FN_

As presented in Figure 2, *B* and *C*, Ldh_FN_ and Bcd_FN_ were also purified as flavoproteins with ƛ_max_ at 385 and 457 nm (Ldh_FN_) and 446 and 374 nm (Bcd_FN_). Heat denaturation of both proteins released the flavin cofactor, which exhibited ƛ_max_ at 374 and 450 nm, diagnostic absorbance maxima of FAD. The calculated extinction coefficients of oxidized FAD bound to Ldh_FN_ and Bcd_FN_ are 9845 M^−1^ cm^−1^ and 12,200 M^−1^ cm^−1^, respectively. A 10% SDS PAGE gel of both purified flavoproteins shows bands at 57 kDa and 40 kDa, which approximate the calculated masses of 54,387 (Ldh_FN_) and 41,206 Da (Bcd_FN_) (Fig. S2).

A structural similarity search of the Protein Data Bank using the Dali Server with an AlphaFold model of the Ldh_FN_ identified the lactate dehydrogenase subunit of Ldh–ETF complex from *A. woodii* (PDB ID: 7QH2) as having the highest structural homology with an RMSD of 1.2 Å over 465 of 467 C⍺ atoms (51% identity and a Z-score = 55.1). The Ldh–ETF complex of *A. woodii* uses flavin-based electron confurcation in which endergonic reduction NAD^+^ to NADH (*E*°′ = −320 mV) by lactate is coupled with the exergonic reduction of NAD^+^ by reduced ferredoxin (*E*°′ ≈ −500 mV) according to the following balanced reaction: lactate + Fd^1+^ + 2 NAD^+^ → pyruvate + Fd^2+^ + 2 NADH (42). The Dali server identified a Bcd from *Megasphaera elsdenii* (Bcd_ME_, PDB ID: 1BUC) as a close structural homolog of the AlphaFold model of Bcd_FN_ with a Z-score of 54.6 and an RMSD of 1.3 Å over 375 of 383 C⍺ atoms (47). As described for *A. fermentans* and *C. kluyveri*, Bcd_ME_ interacts with a bifurcating ETF to couple the endergonic NADH reduction of ferredoxin with the exergonic NADH reduction of crotonyl-CoA (43).

Given the structural similarity between Ldh_FN_ and Bcd_FN_ and homologs of Ldh and Bcd that form complexes with ETF for flavin-based electron bifurcation/confurcation, we used SWISS-MODEL to construct a model of a complex between Ldh_FN_ and ETF_FN_ and between Bcd_FN_ and ETF_FN_, using Ldh-ETF from *A. woodii* (PDB ID: 7QH2) and the Bcd-ETF from *C. difficile* (PDB ID: 5OL2) as templates (21, 40). These two structures were selected because they capture the dehydrogenase and ETF in the resting D (dehydrogenation-connected) state, in which the α-FAD of ETF neighbors the δ-FAD of the dehydrogenase for rapid interflavin electron transfer. As shown in Figs. S3 and S4, the models of Bcd_FN_–ETF_FN_ and Ldh_FN_–ETF_FN_ complexes exhibit a high level of structural similarity to their respective templates. The orientation of ETF_FN_ with respect to the two dehydrogenases likely precludes the formation of a ternary (Ldh_FN_–ETF_FN_–Bcd_FN_) complex.

### Ldh_FN_ pre–steady-state kinetic properties

**Figure 3:**
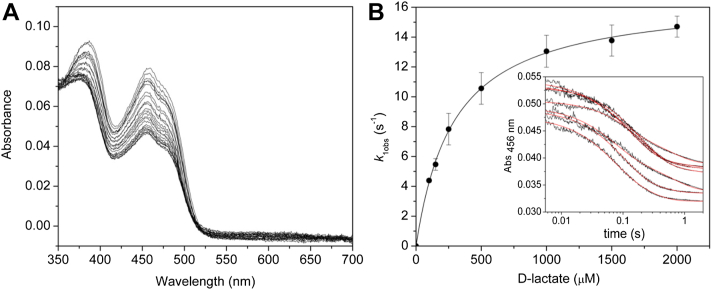
**Pre–steady-state kinetic data associated with D-lactate reduction of Ldh_FN_.***A*, multiwavelength absorbance changes following the rapid mixing of 1 mM D-lactate with an equal volume of 20 μM of Ldh_FN_ in 50 mM potassium phosphate buffer, pH 7.5, at 20 °C under anaerobic conditions as described in Experimental procedures. Spectra were collected over 3 s. *B*, A plot of *k*_1obs_*versus* the concentration of D-lactate. The data were fitted to a hyperbolic function to extract a *K*_d_ of 290 ± 12 μM and a limiting rate constant of reduction (*k*_lim_) of 16.7 ± 2.3 s^−1^. Inset. Single wavelength absorbance traces were collected at 456 nm at varying D-lactate concentrations (0.1–2 mM, post mixing) and fitted to a double-exponential equation to extract *k*_1obs_ and *k*_2obs_. ETF, electron transfer flavoprotein; Ldh, lactate dehydrogenase.

The catalytic preference of Ldh_FN_ for D-lactate *versus* L-lactate was analyzed through pre–steady-state experiments using stopped-flow spectrophotometry. As shown in Figures 3*A* and S5, the rapid mixing of 1 mM D- or L-lactate with 20 μM Ldh_FN_ under anaerobic conditions results in partial reduction of the FAD cofactor. Single wavelength analysis revealed a biphasic decrease in the flavin absorbance maxima following the mixing of 20 μM Ldh_FN_ with D-lactate (0.2–4 mM; Fig. 3*B*). Consequently, a double-exponential equation was used to extract fast (*k*_obs1_) and slow (*k*_obs2_) observed rate constants and their associated amplitudes. Minor deviations were observed in the absorbance values from that of the fitted curve as a result of the small signal-to-noise ratio, which in turn is attributed to partial reduction of Ldh_FN_ by the α-hydroxy acid. The majority (55%) of the amplitude change was associated with the first kinetic phase. A plot of *k*_obs1_ *versus* D-lactate was hyperbolic, and a fit of equation 1 to the data yielded a *K*_D_ of 290 ± 12 μM for D-lactate and a limiting rate constant of reduction (*k*_lim_) of 16.7 ± 2.3 s^−1^ (Fig. 3*B*). Similarly, a plot of *k*_obs2_ *versus* D-lactate was hyperbolic, and a fit of equation 1 to the data yielded a *K*_D_ of 305 ± 44 μM for D-lactate and a limiting rate constant of reduction (*k*_lim_) of 2.1 ± 0.3 s^−1^ (Fig. S5). In contrast, L-lactate reduction of Ldh_FN_ under pseudo-first-order conditions resulted in a monophasic absorbance decrease at 454 nm (Fig. S6). A fit of a single exponential equation generated *k*_obs1,_ and a plot of *k*_obs1_ showed a linear dependence on L-lactate concentration over a range of 0.1 to 2 mM (Fig. S6), implying weak binding of the L-isomer to the active site. Thus, D-lactate is the preferred enantiomer for Ldh_FN_.

### Butyryl-CoA is unable to reduce Bcd_FN_

Anaerobic UV-visible spectroscopy was then used to determine if butyryl-CoA can reduce Bcd_FN_. Although this is not the physiological direction, it can occur, albeit partially, by mass action with the addition of high concentrations of butyryl-CoA. For example, adding 100 μM butyryl-CoA to 13 μM of Bcd_ME_ partially reduced the flavin cofactor (48). Incomplete reduction is attributed to unfavourable thermodynamics associated with the electron transfer, as the butyryl-CoA/crotonyl-CoA couple has a midpoint potential of −10 mV, whereas the δ-FAD/FADH^-^ couple of Bcd_ME_ is −79 mV (49). As shown in Fig. S7, the addition of 200 butyryl-CoA to 15 μM Bcd_FN_ under anaerobic conditions did not shift the absorbance spectra of the Bcd_FN,_ indicating no reduction of the δ-FAD cofactor. These data suggest that the δ-FAD/FADH^-^ couple may be more negative in Bcd_FN_ compared to that of Bcd_ME_.

### Ldh_FN_ steady-state kinetic properties

**Figure 4:**
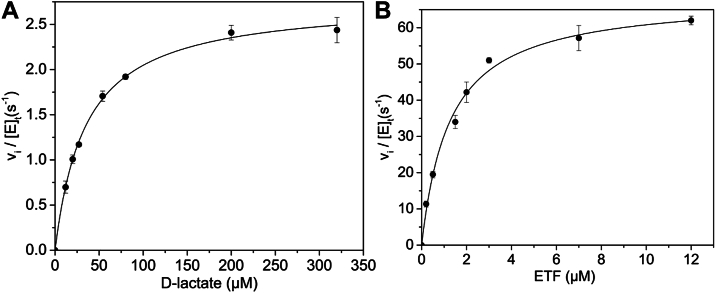
**Ldh_FN_ steady-state turnover assays.***A*, the initial rate of cytochrome c^3+^ reduction was measured in a 1 ml reaction mixture containing 40 nM Ldh_FN_, 100 μM cytochrome c^3+^ and variable concentrations of D-lactate (12–330 μM). The reactions were performed in 50 mM potassium phosphate buffer, pH 7.6, at 20 °C, and the reaction was initiated with the addition of Ldh_FN_. The initial velocity of the reaction divided by the concentration of Ldh_FN_ was plotted as a function of D-lactate concentration. A *k*_cat_ of 2.76 ± 0.04 s^−1^, a *K*_M(D-lac)_ of 35.0 ± 1.6 μM, and a *k*_cat_/*K*_M(D-lac)_ of 2.0 ± 0.2 × 10^4^ M^−1^ s^−1^ were determined by a fit of the data to the Michaelis–Menten equation. *B*, the initial rate of cytochrome c^3+^ reduction was measured in a 1 ml reaction mixture containing 40 nM Ldh_FN_, 100 μM cytochrome c^3+^, 1 mM D-lactate and variable concentrations of ETF_FN_ (1–10 μM). A fit of the Michaelis–Menten equation to the data yielded a *k*_cat_ of 68.5 ± 2.1 s^−1^, a *K*_M(ETF)_ of 1.26 ± 0.13 μM and a *k*_cat_/*K*_M_ of 2.7 ± 1 × 10^7^ M^−1^ s^−1^. For both (*A*) and (*B*), initial rates were determined in triplicate; SDs are indicated by error bars. ETF, electron transfer flavoprotein; Ldh, lactate dehydrogenase.

Given that Ldh_FN_ is specific for D-lactate, the apparent *K*_M_ for D-lactate was determined through steady-state analysis using cytochrome *c*^3+^ as a nonphysiological electron acceptor. The addition of D-lactate to Ldh_FN_ resulted in reduction of cytochrome c^3+^, and the initial rate for this reaction showed a hyperbolic dependence on D-lactate concentration (Fig. 4*A*). A fit of the data to the Michaelis–Menten equation yielded an apparent *k*_cat_ of 2.76 ± 0.04 s^−1^, a *K*_M(D-lac)_ of 35.0 ± 1.6 μM, and a *k*_cat_/*K*_M_ of 7.9 × 10^4^ M^−1^ s^−1^. The cytochrome c^3+^ assay was then used to determine if ETF_FN_ can receive electrons from Ldh_FN_ following D-lactate oxidation. Reaction mixtures containing 40 nM of LDH_FN_, 100 μM of cytochrome c^3+^ and variable concentrations of ETF_FN_ (1–10 μM) were prepared, and the rate of cytochrome c^3+^ reduction was followed with the addition of 1 mM D-lactate. As shown in Figure 4*B*, the initial velocity was hyperbolically dependent on ETF_FN_ concentration, and a fit of the data to the Michaelis–Menten equation generated an apparent *K*_M(ETF)_ of 1.26 ± 0.13 μM, a *k*_cat_ of 68.5 ± 2.1 s^−1^, and a *k*_cat_/*K*_M_ of 2.7 × 10^7^ M^−1^ s^−1^. From these data, we conclude that Ldh_FN_ transfers electrons to ETF_FN_ and that ETF_FN_ is 50-fold faster at electron transfer to cytochrome *c*^3+^ than Ldh_FN_.

### Electron transfer from Ldh_FN_ to ETF_FN_ and Bcd_FN_ to ETF_FN_

**Figure 5:**
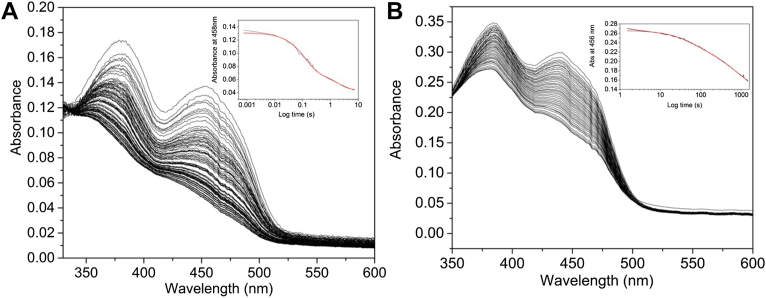
**Electron transfer from Ldh_FN_ to ETF_FN_ and Bcd_FN_ to ETF_FN._***A*, time-resolved optical spectra following the rapid mixing of 20 μM of ETF_FN_ and 20 μM of ETF_FN_ with an equal volume of 1 mM D-lactate. Time-resolved spectral data were acquired over 744 s. Inset: The absorbance trace at 458 nm *versus* time was best fitted to a double exponential, yielding observed rate constants of 7.95 ± 0.18 and 0.55 ± 0.02 s^−1^. *B*, time-resolved optical spectra following the rapid mixing of 20 μM of ETF_FN_ and 20 μM of Bcd_FN_ with an equal volume of 200 μM butyryl-CoA. The spectral data were acquired over 1500 s. Inset. Absorbance trace at 458 nm *versus* time was best fitted to a double exponential, generating observed rate constants of 1.6 ± 0.18 × 10^−2^ s^−1^ and 1.4 ± 0.18 × 10^3^ s^−1^. Electron transfer from Ldh_FN_ to ETF_FN_ and Bcd_FN_ to ETF_FN._*A*, time-resolved optical spectra following the rapid mixing of 20 μM of ETF_FN_ and 20 μM of ETF_FN_ with an equal volume of 1 mM D-lactate. Time-resolved spectral data were acquired over 744 s. Inset: The absorbance trace at 458 nm *versus* time was best fitted to a double exponential, yielding observed rate constants of 7.95 ± 0.18 and 0.55 ± 0.02 s^−1^. *B*, time-resolved optical spectra following the rapid mixing of 20 μM of ETF_FN_ and 20 μM of Bcd_FN_ with an equal volume of 200 μM butyryl-CoA. The spectral data were acquired over 1500 s. Inset. Absorbance trace at 458 nm *versus* time was best fitted to a double exponential, generating observed rate constants of 1.6 ± 0.18 × 10^−2^ s^−1^ and 1.4 ± 0.18 × 10^−3^ s^−1^. Bcd, butyryl-CoA dehydrogenase; ETF, electron transfer flavoprotein; Ldh, lactate dehydrogenase.

Electron transfer from Ldh_FN_ to ETF_FN_ can be conveniently monitored by stopped-flow spectroscopy under strictly anaerobic conditions. Given that ETF_FN_ and Ldh_FN_ likely form a complex, similar to that of the confurcating complex of ETF-Ldh of *A*. *woodii* (40), we rapidly mixed an equimolar solution of Ldh_FN_ (20 μM) and ETF_FN_ (20 μM) with 200 μM D-lactate. As shown in Figure 5*A*, the reaction results in the complete bleaching of the flavin absorbance maxima. The absorbance decrease at 458 nm was biphasic, and a fit of the data to a double-exponential yielded observed rate constants of 7.95 ± 0.18 and 0.55 ± 0.02 s^−1^, with 60% of the amplitude change associated with the first kinetic phase. The time-resolved multiwavelength absorbance did not show transient formation of the red anionic semiquinone, indicating that D-lactate reduction of Ldh_FN_ leads to two-electron reduction of ETF_FN_.

We then determined if Bcd_FN_ could reduce ETF_FN_ in the presence of excess butyryl-CoA. A 1 ml reaction was prepared containing 20 μM ETF_FN_ and 20 μM Bcd_FN_ in 50 mM potassium phosphate, pH 7.6. The time-resolved spectra were then collected following rapid mixing with an equimolar solution of 200 μM butyryl-CoA. As presented in Figure 5*B*, mixing with excess butyryl-CoA does lead to partial reduction of ETF_FN_, without formation of a semiquinone intermediate. However, the reaction is very slow, occurring over ∼30 min. The single-wavelength absorbance traces were biphasic and fit to a double-exponential equation generated the observed rate constants of 1.6 ± 0.18 × 10^−2^ s^−1^ and 1.4 ± 0.18 × 10^−3^ s^−1^.

### Steady-state kinetic properties associated with D-lactate reduction of crotonyl-CoA mediated by Ldh_FN_, Bcd_FN_, and ETF_FN_

**Figure 6:**
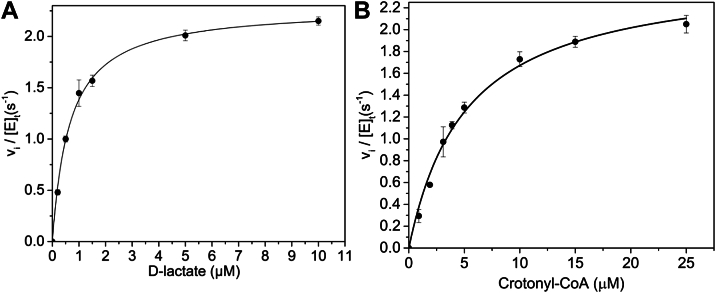
**Ldh_FN_/ETF_FN_/Bdh_FN_ steady-state turnover assays.***A*, the initial rate of NADH oxidation was monitored *via* a coupled assay that involved Ldh_FN_, ETF_FN_, Bcd_FN_, and Ldh_RB_. The 1 ml reactions contained 40 nM ETF_FN_, 40 nM Bcd_FN_, 50 μM crotonyl-CoA, 100 μM NADH, variable concentrations of 0.2 to 10 μM D-lactate, 100 μM NADH, 0.5 mg/ml of Ldh_RB_ in 50 mM potassium phosphate buffer pH 7.5. The reaction was initiated with the addition of 40 nM Ldh_FN_ and the absorbance at 340 nm at 20 °C was recorded over a select time domain. The initial velocity was divided by the concentration of Ldh_FN_, and the observed rate constant (*k*_obs_) was plotted as a function of D-lactate concentration, which yielded a *k*_cat_ of 2.29 ± 0.04 s^−1^, a *K*_M(D-lac)_ of 0.65 ± 0.04 μM and a *k*_cat_/*K*_M_ of 1.75 ± 0.05 × 10^6^ M^−1^ s^−1^. *B*, same as for *panel A*, except the 1 ml reactions contained 40 nM ETF_FN_, 40 nM Bcd_FN_, 1 mM D-lactate, 100 μM NADH and a variable concentration of crotonyl-Coa (1–25 μM), 100 μM NADH, 0.5 mg/ml of Ldh_RB_ in 50 mM potassium phosphate buffer pH 7.6. The reaction was initiated by adding 40 nM Ldh_FN_, and the absorbance change was followed at 340 nm at 20 °C. The initial velocity was divided by the concentration of Ldh_FN_, and the observed rate constant (*k*_obs_) was plotted as a function of crotonyl-CoA concentration, which yielded a *k*_cat_ of 2.54 ± 0.09 s^−1^, a *K*_M(crotonyl-CoA)_ of 5.2 ± 0.5 μM, and a *k*_cat_/*K*_M_ 4.9 ± 0.2 × 10^5^ M^−1^ s^−1^. For both (*A*) and (*B*), initial rates were determined in triplicate; SDs are indicated by error bars. Bcd, butyryl-CoA dehydrogenase; ETF, electron transfer flavoprotein; Ldh_RB_, lactate dehydrogenase from rabbit muscle.

The coupling of D-lactate oxidation with the reduction of crotonyl-CoA by Ldh_FN_, Bcd_FN_, and ETF_FN_ was measured through a coupled spectrophotometric assay using lactate dehydrogenase from rabbit muscle (Ldh_RB_), which catalyzes the NADH-dependent reduction of pyruvate to L-lactate. The coupled reaction was performed under anaerobic conditions as described in the experimental methods. The 1 ml reactions contained 40 nM ETF_FN_, 40 nM Bcd_FN_, 50 μM crotonyl-CoA, 100 μM NADH, 0.5 mg/ml of Ldh_RB_, and variable concentrations of 0.2 to 10 μM D-lactate in 50 mM potassium phosphate buffer, pH 7.5. The reaction was initiated with the addition of 40 nM Ldh_FN,_ and the absorbance change was monitored at 340 nm at 20 °C. An absorbance decrease at 340 nm (indicating NADH oxidation) was observed if all reaction mixture components were present. As shown in Figure 6*A*, NADH oxidation was hyperbolically dependent on D-lactate concentration, and a fit of the data to the Michaelis–Menten equation yielded a *k*_cat_ of 2.29 ± 0.04 s^−1^, a *K*_M(D-lac)_ of 0.65 ± 0.04 μM, and a *k*_cat_/*K*_M_ of 1.75 ± 0.05 × 10^6^ M^−1^ s^−1^. The experiment was repeated, except D-lactate concentration remained constant at 50 μM, while the crotonyl-CoA concentration varied from 1 to 25 μM. As shown in Figure 5*B*, the hyperbolic data were fitted to the Michaelis–Menten equation, yielding a *k*_cat_ of 2.54 ± 0.09 s^−1^, a *K*_M(crotonyl-CoA)_ of 5.2 ± 0.5 μM, and a *k*_cat_/*K*_M_ of 4.9 ± 0.2 × 10^5^ M^−1^ s^−1^.

The reverse reaction (butyryl-CoA + pyruvate → lactate + crotonyl-CoA) by Bcd_FN_/Ldh_FN_/ETF_FN_ was then measured through a second coupled spectrophotometric assay using D-lactate dehydrogenase from *Lactobacillus leichmannii* (MilliporeSigma; Ldh_LL_). A 1 ml reaction was prepared containing 0.5 mg/ml Ldh_LL_, 120 μM pyruvate, 100 μM NAD^+^, and 40 nM of ETF_FN_, Ldh_FN,_ and Bcd_FN_ in 50 mM potassium phosphate buffer, pH 7.6. The reaction was initiated with the addition of 200 μM butyryl-CoA. We did not observe an absorbance increase at 340 nm, which indicated that Bcd_FN_/Ldh_FN_/ETF_FN_ is unable to catalyze the reverse reaction, the butyryl-CoA–dependent reduction of pyruvate, at least under the experimental conditions tested.

We also took the opportunity to measure the lactate oxidase activity of the Ldh_FN_ in the presence of ETF_FN_ and Bcd_FN_. A 1 ml reaction was prepared containing 100 μM NADH, 0.5 mg/ml of Ldh_RM_, and 100 nM Ldh_FN_ in 50 mM potassium phosphate buffer, pH 7.6. The addition of 1 mM D-lactate resulted in oxidation of NADH at a turnover rate of 1.6 s^−1^. The addition of 100 nM ETF_FN_ or 100 nM ETF_FN_ and 100 nM Bcd_FN_ to the reaction mixture did not result in a change in the turnover rate, indicating that the oxidase activity was primarily associated with Ldh_FN_ oxidation of D-lactate.

### Redox potentiometry

**Figure 7:**
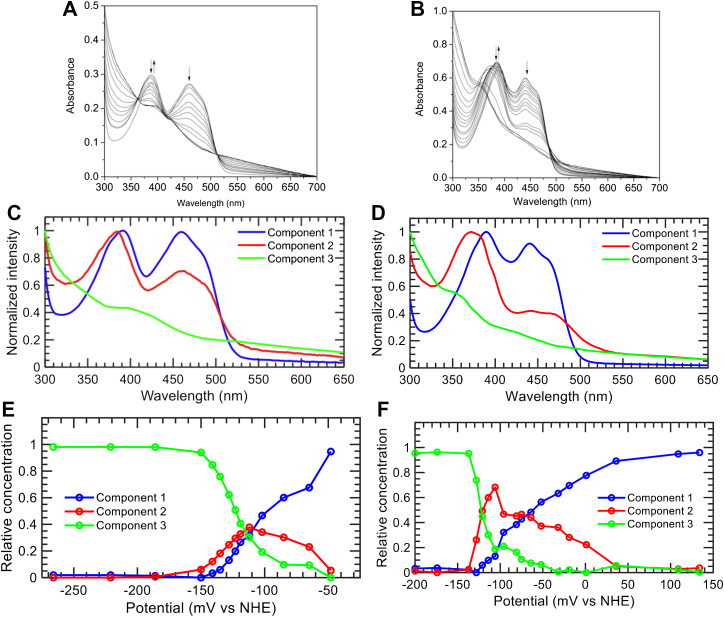
**Redox titrations.** Absorption spectra for the redox titration of (*A*) 50 μM Ldh_FN_ (*B*) ETF_FN_. The spectra were obtained after each addition of dithionite as described in the Experimental procedures. Deconvoluted spectra of the intermediates resolved from potential-dependent global MCR-ALS analysis of the data shown in *panels A* (*C*) and *B* (*D*). The relative concentrations of the extracted intermediates for Ldh_FN_ (*E*) and ETF_FN_ (*F*) as a function of potential. Bcd, butyryl-CoA dehydrogenase; ETF, electron transfer flavoprotein; Ldh, lactate dehydrogenase; MCR-ALS, multivariate curve resolution-alternating least squares.

The thermodynamics underpinning the electron transfer from Ldh_FN_ → ETF_FN_ → Bcd_FN_ was then investigated by measuring the reduction potentials of each of the FAD cofactors. Each flavoprotein was sequentially reduced with dithionite in an anaerobically maintained glovebox. Throughout the titration, UV-visible spectra of the flavoproteins were acquired at a specified reduction potential. Chemical reduction of Bcd_FN_ resulted in bleaching of the flavin absorbance maxima, 456 nm. Unfortunately, Bcd_FN_ proved to be particularly unstable during the redox titration, and the significant increase in light scattering prevented the determination of the reduction potential. Figure 7 shows the absorbance spectra of the Ldh_FN_, and ETF_FN_ throughout the titration. Early in the titration, the δ-FAD of Ldh_FN_ and α-FAD of ETF_FN_ form the red anionic semiquinone as evidenced by the formation of a peak at 380 nm and a decrease at 456 nm. Further reduction of Ldh_FN_ and ETF_FN_ resulted in a decrease at 380 and 456 nm, reflecting conversion of the red anionic semiquinone to the hydroquinone.

Due to small absorbance shifts at 380 nm, the entire spectral data set was analyzed by the multivariate curve resolution-alternating least squares (MCR-ALS) algorithm (50). MCR is considered a soft modeling approach as no *a priori* assumptions are made on the underlying physical model. In other words, the system is not constrained to a particular kinetic or redox interpretation. We determined the appropriate number of components to be included by singular value decomposition, which revealed three components for both Ldh_FN_ and ETF_FN_ (51). The initial spectra estimates were done using the purest variable detection method. Optimization by ALS was performed with the constraints that the spectra and concentrations are nonnegative over the entire dataset. Figure 7, *C* and *D* show the extracted spectra for the three components are in good agreement with those expected for FAD (quinone), FAD^•-^ (red anionic semiquinone) and FADH^-^ (anionic hydroquinone) forms of the cofactor, with the caveat that components 2 and 3 also encompass the spectral changes, or light scattering, attributed to precipitation of Ldh_FN_ and ETF_FN_ during the course of the titration. The relative concentrations extracted are in line with the expected Nernstian redox behaviours for one-electron processes, supporting the results of the soft modeling. The redox potential of the FAD/FAD^•-^ couple is extracted from the crossovers of the components 1 and 2 traces, while the redox potential of the FAD^•-^/FADH^-^ couple is extracted from the crossover of components 2 and 3 traces. This yielded −70 mV and −122 mV for the FAD/FAD^•-^ and FAD^•-^/FADH^-^ of ETF_FN_, and more closely spaced potentials of −109 mV and −115 mV for the corresponding Ldh_FN_ redox couples. The amount of semiquinone generated during the titration is higher in ETF_FN,_ with relative concentrations of ∼ 0.45 at the midpoint potentials, than ∼0.36 for Ldh_FN_.

## Discussion

In 1964, Baldwin and Milligan reported that an ETF from *M. elsdenii* (ETF_ME_) could transfer electrons *via* NADH to Bcd for the reduction of crotonyl-CoA to butyryl-CoA (52). In the same study, a pyridine nucleotide-independent lactate dehydrogenase (Ldh_ME_) was partially purified. Although not experimentally demonstrated, the authors suggested that lactate oxidation could provide reducing equivalents for the reduction of crotonyl-CoA. Purification of the Ldh_ME_ by Brockman *et al.*, 11 years later, revealed that it contained an FAD cofactor, was specific for D-lactate and was able to transfer electrons *via* an ETF (ETF_ME_) to a Bcd_ME_ for conversion of crotonyl-CoA to butyryl-CoA (53). Notably, NADH could substitute for lactate in the reaction because the ETF_ME_ is a bifurcating ETF, as confirmed by Chowdhury *et al.* in 2015 (43). Herein, we established that ETF_FN_, like ETF_ME_, also transfers electrons from Ldh_FN_ to Bcd_FN_, enabling the direct coupling of lactate oxidation to butyryl-CoA formation. However, unlike ETF_ME_, ETF_FN_ is a canonical ETF and is encoded in the same gene cluster as genes for Ldh_FN_ and Bcd_FN_. *M*. *elsdenii* contains two sets of genes encoding for the α- and β-subunits of ETF, but neither set is encoded in an *lct* gene cluster, analogous to those shown in Figure 1.

### Kinetic analysis of Ldh_FN_-ETF_FN_-Bcd_FN_

Herein, we demonstrated through pre–steady-state kinetic analysis that Ldh_FN_ is stereoselective for the D-isomer of lactate, with the hydroxy acid exhibiting a *K*_D_ of 290 ± 12 μM and a limiting rate constant of reduction (*k*_lim_) of 16.7 ± 2.3 s^−1^. As shown for Ldh_ME_, rapid mixing of Ldh_FN_ with excess D-lactate resulted in multiphasic reduction of the flavin cofactor, which may be attributed to the enzyme existing in more than one catalytically competent form (54). Notably, steady-state experiments revealed that the initial velocity associated with the D-lactate–dependent reduction of cytochrome *c*^3+^ in the presence of Ldh_FN_ and ETF_FN_ is hyperbolically dependent on the concentration of the latter protein, with the overall reaction exhibiting a *k*_cat_ of 68.5 ± 2.1 s^−1^. This turnover number is 4-fold higher than the limiting rate of reduction of Ldh_FN_ by D-lactate 16.7 s^−1^, which suggests that ETF_FN_ and Ldh_FN_ form a complex similar to that of a confurcating Ldh–ETF complex, as shown in Fig. S4. The slightly more positive redox potentials associated with α-FAD of ETF_FN_ likely accelerate the rate of reduction by D-lactate and lead to more complete reduction of the flavin cofactors.

For the overall reaction of lactate + crotonyl-CoA → pyruvate + butyryl-CoA, *K*_M_ values in the submicromolar and micromolar range were recorded for the two substrates (*K*_M (D-lactate)_ of 0.65 ± 0.04 μM and *K*_M(crotonyl-CoA)_ of 5.2 ± 0.5 μM), resulting in catalytic efficiencies of 1.75 ± 0.05 × 10^6^ M^−1^ s^−1^ (D-lactate) and 4.9 ± 0.2 × 10^5^ M^−1^ s^−1^ (crotonyl-CoA) at 25 °C. Based on these values, the Ldh_FN_–ETF_FN_–Bcd_FN_ redox pathway is primed to operate at low intracellular concentrations of D-lactate and crotonyl-CoA. Importantly, the reverse reaction (pyruvate + butyryl-CoA → lactate + crotonyl-CoA) does not occur. We speculate that the reverse reaction is prevented—in part—by the lower reduction potential of the δ-FAD cofactor of Bcd_FN_, at least in comparison to other Bcd homologs (55). Although we were not able to determine Bcd_FN_ reduction potential due to the instability of protein during the reduction potential, we observed that butyryl-CoA, even at a concentration of 200 μM, was unable to reduce the δ-FAD cofactor of Bcd_FN_. Moreover, while excess butyryl-CoA did lead to partial flavin reduction of an equimolar solution of Bcd_FN_ and ETF_FN_, the reaction was very slow (>30 min) and thus not physiologically relevant. These data suggest that the reduction potential of δ-FAD/δ-FADH^-^ of Bcd_FN_ is more negative than the corresponding couple in Bcd_ME_ (49). Straight-chain acyl-CoA dehydrogenases typically contain a tyrosine residue (Tyr361; numbering of Bcd from *C. difficile*) in the substrate-binding cleft (47, 56, 57). As shown in Fig. S8, the phenolic group of this side chain is nearly perpendicular to the *re*face of the flavin, a position likely stabilized through hydrogen bond interaction with the side chain of Gln (Gln248). In Bcd_FN_, this conserved tyrosine is replaced by phenylalanine (Phe360), and the glutamine is replaced by isoleucine (Ile247). Replacement of the phenolic group with a less electron-rich phenyl group may change the nature of the aromatic interaction with reduced flavin, which in turn would influence the δ-FAD/δ-FADH^-^ reduction potential (55).

### Comparison of lct gene clusters

**Figure 8:**
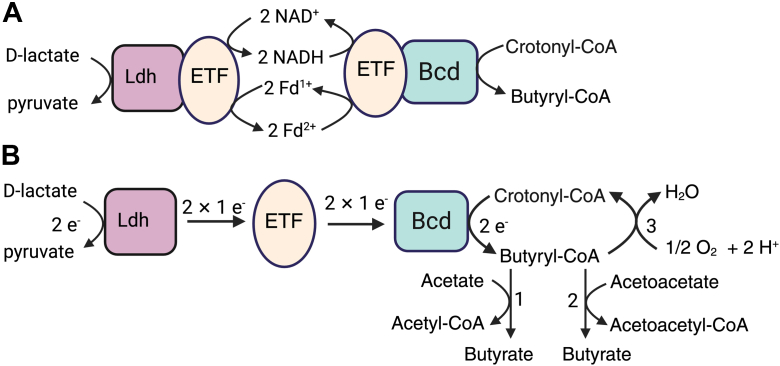
**Canonical *versus* bifurcating ETF.***A*, based on sequence alignment, the ETF encoded in the *lct* gene cluster of *Anaerobutyricum hallii* may function as a confurcating ETF in complex with Ldh and a bifurcating ETF with Bcd. The overall reaction (lactate + crotonyl-CoA → pyruvate + butyryl-CoA) would lead to stoichiometric recycling of NAD/NADH and ferredoxin (Fd). *B*, in *Fusobacterium nucleatum*, ETF functions as a canonical ETF transferring two electrons from Ldh to Bcd. In *A. hadrus*, *A. caccea* and *C. catus, A. hallii,* the conversion of lactate and acetate to butyrate may occur through the combined action of Ldh/ETF/Bcd and butyrate-acetate CoA-transferase (denoted by 1) and in *F. nucleatum*, the release of butyrate is dependent on butyrate-acetoacetate CoA-transferase (denoted by 2). In *F. nucleatum*, butyryl-CoA oxygen oxidoreductase (denoted by 3) can oxidize butyryl-CoA to crotonyl-CoA transferring reducing equivalents to a diiron center for reduction of O_2_ to H_2_O. Bcd, butyryl-CoA dehydrogenase; ETF, electron transfer flavoprotein; Ldh, lactate dehydro genase.

As mentioned in the Introduction, the organization of the Ldh_FN_, Bcd_FN_, and EtfAB_FN_ genes in *F. nucleatum* is similar to that of *lct* gene clusters of *A. hadrus*, *A. caccae*, *A. hallii*, and *C. catus.* These species of bacteria belong to the Lachnospiraceae family of the phylum Firmicutes and are noted for their ability to utilize lactate as an energy source (25, 26). A notable difference in their *lct* gene clusters is the presence of genes encoding a lactate permease, a lactate racemase, and, in some cases, a transcriptional regulatory protein (58). *A. hallii*, *A. hadrus,* and *A. caccae* were shown to produce butyrate from lactate, with net consumption of acetate (26, 59). Ducan *et al.* proposed that the pyruvate generated from Ldh was rerouted to butyryl-CoA *via* the action of pyruvate ferredoxin oxidoreductase and the production of acetyl-CoA, as illustrated in Fig. S9. However, based on our results, we suggest a simpler scheme involving direct conversion of lactate to butyryl-CoA *via* the Bcd/ETF/Ldh pathway (Fig. 8). The release of butyrate occurs through a butyryl-CoA:acetate CoA transferase reaction, which consumes one equivalent of acetate. *F. nucleatum* does not encode a homolog for this enzyme, but does encode a butyryl-CoA:acetoacetate transferase, which transfers the CoA moiety from butyryl-CoA to acetoacetate (generated through the lysine fermentation pathway), yielding butyrate and acetoacetyl-CoA.

A sequence alignment of the etfB subunit encoded in the *lct* gene clusters of *A. hadrus*, *A. caccea,* and *C. catus,* indicates that the ETF encoded in these gene clusters is canonical. However, *A. hallii* appears to encode a bifurcating in its *lct* gene cluster as it retains the conserved glycine residue (Fig. S10). It is plausible that a bifurcating ETF is still able to couple lactate oxidation with butyryl-CoA formation, with the caveat that the ETF transiently associates with the Ldh for electron confurcation and then Bcd for electron bifurcation. The overall reaction, as shown in Figure 8, would involve recycling of ferredoxin and NAD^+^/NADH. The difference in canonical *versus* bifurcating ETFs within the *lct* gene clusters also coincides with differences in gene order. Specifically, the Bcd gene precedes the EtfAB genes in all canonical ETFs, where it is encoded downstream of *EtfAB* in the putative bifurcating system of *A. hallii*. Differences in gene organization suggest that *EtfAB* was co-opted twice to couple lactate oxidation with the formation of butyryl-CoA.

### Canonical *versus* bifurcating ETF

One could ask whether there is a physiological advantage to a canonical *versus* a bifurcating ETF in the coupling of lactate oxidation with butyryl-CoA formation. A bifurcating ETF appears superfluous in this metabolic context, given that the overall reaction is thermodynamically favorable. The overall reaction leads to the recycling of the low-potential reductant (flavodoxin or ferredoxin) and the pyridine nucleotide. The deployment of a canonical ETF in this particular pathway may be a way to avoid the formation of superoxide (O_2_^•-^) and hydrogen peroxide, the latter of which can react with labile ferrous iron to form the hydroxyl radical •OH. Work by Chowdury *et al.* showed that in the presence of O_2_, the Bcd-ETF_ME_ can function as a NADH oxidase, whereby O_2,_ instead of ferredoxin, accepts electrons from the β-FAD following reduction by NADH. Perhaps swapping the β-FAD for a redox-inert AMP in ETF_FN_ may be a way to safeguard against the formation of reactive oxygen species. As a common constituent of the oral cavity and invasive organisms of extra-oral sites (*e.g.,* tumors, adenomas, amniotic fluid), *F. nucleatum* likely encounters transient exposure to O_2_, and deployment of a canonical ETF for the metabolism of lactate may be an adaptation for survival in its ecological niches. We measured the propensity of the Ldh_FN_/ETF_FN_/Bcd_FN_ system to donate electrons to O_2_ in the presence of excess lactate, noting that the lactate oxidase activity was solely attributed to Ldh_FN._ The addition of ETF_FN_ and Bcd_FN_ did not accelerate the lactate oxidase activity, indicating that they are not the primary source of reactive oxygen species.

### Thermodynamic properties of ETF_FN_

Redox titrations of ETF_FN_ showed that, similar to other ETFs, the protein stabilizes the red anionic semiquinone. However, the reduction potentials (α-FAD/α-FAD•^-^ = −70 mV and an α-FAD•^-^/α-FADH^-^ = −122 mV) are more negative and more closely spaced compared to other canonical and bifurcating ETFs. For example, the bifurcating ETF_ME_, exhibits a positive *E*°′ for α-FAD/α-FAD•^-^ of +81 mV and an *E*°′ for α-FAD•^-^/α-FADH^-^ of −136 mV (217 mV separation) (22). The elevated potential of the α-FAD/α-FAD•^-^ couple of ETF_ME_ indicates that the resting form of ETF_ME_ *in vivo* is with the cofactor in the α-FAD•^-^ redox state, poised to accept a single electron from β-FADH^-^. This accommodates flavin-based bifurcation, as α-FAD•^-^ is precluded from accepting two electrons from β-FADH^-^, which in effect forces the second electron on the ensuing β-FAD•^-^ to reduce ferredoxin/flavodoxin. Likewise, the canonical ETF from *M. methylotropus* (ETF_MM_) exhibits a wide separation between α-FAD/α-FAD•^-^ (+196 mV) and α-FAD/α-FAD•^-^ (−197 mV) to the extent that there is quantitative formation of α-FAD•^-^ in the reductive titration of ETF_MM_ (60). By comparison, the maximum relative concentration of α-FAD•^-^ in the ETF_FN_ titration was 0.45. The extremely positive value for the first reductive step of the α-FAD of ETF_MM_ coupled with second reductive step occurring extremely slowly, strongly suggests that the α-FAD of ETF_MM_ cycles between the α-FAD and α-FAD•^-^. Conversion between these redox forms accommodates the biological role of ETF_MM_: accepting a single electron from the iron-sulfur cluster of trimethylamine dehydrogenase (60).

In contrast, a narrow separation of the two-reduction potentials of α-FAD in ETF_FN_, and more negative *E*°′ values, primes the flavoprotein to accept two electrons from Ldh_FN_. Indeed, rapid mixing of an Ldh_FN_ and ETF_FN_ in the presence of excess D-lactate shows a complete bleaching of the flavin absorbance spectra, indicating full reduction of both flavoproteins without transient formation of a semiquinone species. In this capacity, ETF_FN_ is more similar to the canonical ETFs of the mitochondria and *P. denitrificans,* with the exception that the corresponding potentials for these ETF homologs are more positive in comparison to that of ETF_FN_, and thus primed to accept electrons from acyl-CoA dehydrogenases (61, 62).

A model of ETF_FN_ reveals that it contains a conserved arginine residue (αR237) that lies perpendicular to the xylene portion of the isoalloxazine ring, a conserved serine (αS254) that forms a hydrogen bond to the N5 of the cofactor and a histidine (αH274) (Fig. S11). All three of these residues have been shown in canonical and bifurcating ETFs to contribute to the high *E*° associated with the α-FAD/α-FAD•^-^ couple, and thus the tendency to cycle between α-FAD•^-^ and α-FADH^-^ (41, 63, 64, 65, 66). Thus, the structural rationale for the closely spaced and more negative α-FAD/α-FAD•^-^ couple in ETF_FN_ is not evident from the constellation of residues surrounding the cofactor. It is conceivable that more distal residues and/or the conformational properties of the ETF_FN_ influence the redox properties of the cofactor.

### Maintaining redox balance during oxidative stress

As noted in the introduction, the *lct* operon is regulated by ModRS, a two-component regulatory system that serves as a global regulator of the oxidative stress response (38). Under oxidative stress, ModRS upregulates genes involved in repairing oxidatively damaged proteins (*e.g.,* methionine sulfoxide reductase), and metabolic pathways that function to offset the inactivation of the central metabolic enzyme, pyruvate formate lyase (67, 68, 69). The upregulation of the *lct* cluster may be a strategy for increasing cellular concentrations of butyryl-CoA, which can be used by butyryl-CoA oxygen oxidoreductase (BOOR; FN1423-FN1424; Fig. 8). BOOR catalyzes the oxidation of butyryl-CoA to crotonyl-CoA and shuttles reducing equivalents to a diiron motif for the reduction of O_2_ to H_2_O (70). The product, crotonyl-CoA, can be reduced by Ldh_FN_/ETF_FN_/Bcd_FN_ for further lactate conversion to pyruvate or serve as a terminal electron acceptor for flavin-based electron bifurcation by Bcd-ETF (FN0783-FN0785).

In several *Clostridia* species (*e.g., Clostridium ljungdahlii* and *Clostridioides difficile*), O_2_ is detoxified by an NAD(P)H-dependent oxygen oxidoreductase, which, as the name suggests, uses NAD(P)H to reduce O_2_ to H_2_O (71, 72). Studies of *C. ljungdahlii* have shown that O_2_ exposure not only increases the expression of NAD(P)H-dependent O_2_ reduction enzymes but also metabolic enzymes that produce NAD(P)H or reduced ferredoxin, which can be used to form more reduced pyridine nucleotides (73). These enzymes include the carbon monoxide dehydrogenase complex that catalyzes the oxidation CO to CO_2_, glyceraldehyde 3-phosphate dehydrogenase that catalyzes the NAD^+^-dependent oxidation of glycerol 3-phosphate to glyceraldehyde 3-phosphate, and the G-subunit of the RNF complex, which reduces NAD^+^ (73). Likewise, O_2_ exposure in *C. difficile*, which also uses NAD(P)H-dependent O_2_ detoxifying system, resulted in suppression of the reductive fermentation pathways for proline and glycine, which contribute to the formation of NAD^+^, and activation of the oxidative fermentation pathway for branch-chain amino acids, which generate NADH (74). Thus, the upregulation of the *F*. *nucleatum lct* gene cluster to mitigate O_2_ exposure parallels that observed in other anaerobes. The role of LrgAB in this *lct* gene cluster is a topic for future studies, but we speculate that it functions to transport byproducts of carbohydrate metabolism (*e.g.,* pyruvate and/or lactate) as part of its adaptation to oxidative stress, as shown for other bacteria (30, 31, 75).

### Conclusion

In summary, this study reveals that *F. nucleatum* employs a canonical ETF to directly couple lactate oxidation to butyryl-CoA formation. The Ldh_FN_-ETF_FN_-Bcd_FN_ system is tuned for two successive one-electron transfers between the FAD cofactors associated with each of the three redox proteins. ETF in particular exhibits closely spaced and negative redox potentials that support the flavin cofactor cycling between the oxidized and hydroquinone state under physiological conditions. Similar *lct* gene clusters in other anaerobes appear to function in the same capacity, but some species may employ a combination of flavin-based confurcation/bifurcation. We speculate that the canonical pathway avoids NADH oxidase activity and O_2_^•-^ and hydrogen peroxide formation associated with bifurcating ETFs. ModRS upregulation of the *lct* gene cluster may be an adaptive mechanism for maintaining redox balance under oxidative stress, paralleling responses seen in other anaerobes. Future research into LrgAB may further expand on its emerging role in the transport of small metabolites of carbohydrate metabolism as a mechanism for maintaining redox balance in response to oxidative stress.

## Experimental procedures

### Materials

The synthesis of butyryl-CoA and crotonyl-CoA was performed by acylation of CoA in aqueous 1 M KHCO_3_ using 1 M butyric anhydride and 1 M crotonic anhydride in acetonitrile at a slight molar excess. Following acidification, the CoA thioester was purified on a C18 column and stored as a lyophilized powder at −70 °C. Restriction enzymes and Q5 DNA polymerase were purchased from NEB BioLabs. Oligonucleotides were ordered from Integrated DNA Technologies. CoA, Ldh_RB_, and a D-lactate dehydrogenase from *L. leichmannii* (Ldh_LL_) were from MilliporeSigma. All other chemicals were purchased from Thermo Fisher Scientific.

### Plasmid construction and recombinant protein expression

The cDNA corresponding to the ETF_FN_ (FN1533/34), Bcd_FN_ (FN1535), and Ldh_FN_ (FN1536) were amplified from the genomic DNA of *F. nucleatum* subsp. *polymorphum* ATCC 10953 using Q5 DNA polymerase. The forward and reverse primers for amplifying the genes are provided in Table S1. The PCR products corresponding to ETF_FN_, Ldh_FN_, and Bcd_FN_ were digested with *Nde*I and *Bam*HI and subcloned into the *Nde*I/*Bam*HI site of pet28a, fusing a thrombin-cleavable His_6_-tag onto the N terminus of each protein. The three constructs (pet28-Ldh, pet28-EtfAB, pet28-Bcd) were confirmed by sequencing and were transformed with *E. coli* BL21(DE3) pLysS. Cells harboring the plasmids were selected on Luria–Bertani plates containing 50 μg/ml of kanamycin. The transformed cells were grown at 37 °C at 200 rpm in 100 ml of Luria–Bertani broth supplemented with 50 μg/ml of kanamycin for 16 h. Ten millilitres of the overnight culture were then used to inoculate 1 L of 2 YT medium. At an absorbance (600 nm) of ∼0.6, 0.2 mM IPTG was added to the cultures to induce gene transcription. Following induction, the cultures continued to grow at 25 °C and 200 rpm for an additional 16 h. The cells were then harvested by centrifugation, frozen, and stored at −70 °C.

### Protein purification

Bcd_FN_, ETF_FN_, and Ldh_FN_ were purified by the same protocol, except where noted. All steps in the protein purification process were performed on ice or at 4 °C. Approximately 15 g of cell pellet was resuspended in buffer A (50 mM potassium phosphate, pH 7.4, 0.15 M NaCl) with 20 mM imidazole, 1 mM benzamidine, and 1 mM phenylmethylsulfonyl fluoride. Cells were lysed, and the genomic DNA was sheared through sonication. The cell lysate was clarified by centrifugation (40,000*g* for 60 min). Then, the supernatant was applied to a 5 ml fast-flow Ni-NTA column (Cytiva) that was equilibrated with buffer A containing 20 mM imidazole. For the preparation of Bcd_FN_, 200 μM of FAD was added to the crude extract before application to the Ni-NTA column. The column was then washed with 10 column volumes of buffer A supplemented with 20 mM imidazole, followed by five column volumes of buffer A with 40 mM imidazole. The flavoproteins were then eluted with buffer A containing 300 mM imidazole. The eluted protein was dialyzed against 50 mM potassium phosphate, pH 7.4, 0.15 M NaCl for 16 h.

The concentration of all protein samples was determined using the bicinchoninic acid kit (MilliporeSigma) with bovine serum albumin as the standard. The flavin extinction coefficient associated with each protein was determined by recording the electronic absorption spectrum of the flavoprotein, and then heat-denaturing the protein sample and removing the precipitated protein by centrifugation (76). The absorption spectrum of the released flavin was then recorded, and an extinction coefficient of 11,300 M^−1^ cm^−1^ for free FAD was used to calculate the extinction coefficient of the flavoprotein.

### Pre–steady-state kinetic analysis

All pre–steady-state kinetic experiments were performed under anaerobic conditions using an SF-61DX2 stopped-flow device (TGK Scientific) with the sampling handling unit placed in an anaerobically maintained glovebox from Belle Technology. A photodiode array detector was used to monitor changes in the flavin spectra (300–700 nm) over a selected time domain. In contrast, a photomultiplier tube was used to follow changes at a single wavelength. Buffer (50 mM potassium phosphate, pH 7.5) was made anaerobic by purging extensively with N_2_ (99.998%) for 2 h before introduction into the glovebox. Lyophilized powders of the substrates, L-lactate, D-lactate, and butyryl-CoA, were introduced into the glovebox and dissolved in an anaerobic buffer. Concentrated (100–200 μM) 1 ml aliquots of proteins (Ldh_FN_, ETF_FN_, and Bcd_FN_) were introduced into the glovebox and were left uncapped in a 4 °C fridge in the glovebox for 16 h before use. Reduction of Ldh_FN_ by L-lactate or D-lactate was performed by rapidly mixing a 20 μM solution of oxidized Ldh_FN_ with varying concentrations of either stereoisomer. The absorbance traces acquired at 456 or 458 nm were fit to a single- or double-exponential equation using the data fitting software Kinetic Studio (TgK Scientific). The resulting observed rate constants (*k*_obs_) were plotted as a function of L-lactate or D-lactate concentrations. Equation 1 was used to fit hyperbolic data, where *k*_lim_ is the limiting rate constant for reduction and *K*_D_ is the dissociation constant.

$$k_{obs}=\frac{k_{\lim}\left[D-lactate\right]}{K_{D}+\left[D-lactate\right]}$$

Anaerobic stopped-flow spectroscopy was used to determine if the ⍺-FAD cofactor of ETF_FN_ was able to receive electrons from Ldh_FN_ or Bcd_FN_ following reduction by their respective substrates, D-lactate and butyryl-CoA. These electron transfer reactions were performed in an anaerobically maintained glovebox in 50 mM potassium phosphate, pH 7.5. For Ldh_FN_ reduction of ETF_FN,_ a solution of 20 μM of ETF_FN_ and 20 μM of Ldh_FN_ was rapidly mixed with 200 μM D-lactate, and for Bcd_FN_ reduction of ETF_FN,_ a solution of 20 μM of ETF_FN_ and 20 μM of Bcd_FN_ was rapidly mixed with 200 μM butyryl-CoA. The time-resolved spectra were recorded with a photodiode array detector over a select time domain.

### Steady-state kinetic analysis

Macroscopic kinetic parameters (apparent *K*_M_ for D-lactate and ETF_FN_) were determined under steady-state conditions using cytochrome *c*^3+^ as a final and nonphysiological electron acceptor. The rate of Ldh_FN_-catalyzed reduction was determined at 25 °C by following the absorbance change at 550 nm (Δε = 21.1 mM^–1^ cm^−1^) on a Lambda 25 UV-visible spectrometer (PerkinElmer). The 1 ml reaction mixture contained 50 mM potassium phosphate pH 7.5, 100 μM cytochrome *c*^*3*+^, and variable concentrations of D-lactate (12–320 μM). The reactions were initiated with the addition of 40 nM of Ldh_FN_. Initial velocities were plotted against substrate concentrations to extract the apparent *K*_M_ for D-lactate. The cytochrome c^3+^ assay was also used to determine if ETF_FN_ can receive electrons from Ldh_FN_ following D-lactate oxidation. Reaction mixtures contained 40 nM of LDH_FN_, 100 μM of cytochrome c^3+^ and variable concentrations of ETF_FN_ (1–10 μM). The rate of cytochrome c^3+^ reduction was followed with the addition of 1 mM D-lactate. Absorbance changes were observed at 550 nm, and the initial velocity of the reaction was calculated using an extinction coefficient of 8.3 mM^−1^ cm^−1^.

### Coupled assays

The D-lactate–dependent reduction of crotonyl-CoA and the butyryl-CoA reduction of pyruvate by Ldh_FN_/ETF_FN_/Bcd_FN_ were measured through a coupled spectrophotometric assay involving L-Ldh_RB_ or a D-lactate dehydrogenase from *L. leichmannii* (Ldh_LL_). The reactions were performed under anaerobic conditions in the glovebox. For the stopped-flow experiments, the flavoproteins Ldh_LL_ and Ldh_RB_ (in 1 ml aliquots) were introduced into the glovebox and allowed to equilibrate with the N_2_ atmosphere for 16 h at 4 °C. The buffer (50 mM potassium phosphate, pH 7.5) was prepared anaerobically as described for the stopped-flow studies. Powdered forms of crotonyl-CoA, butyryl-CoA, lactate, and pyruvate were brought into the glovebox and dissolved in the anaerobic buffer. For the “forward” reaction (lactate + crotonyl-CoA → pyruvate + butyryl-CoA), a 1 ml reaction was prepared containing 40 nM ETF_FN_, 40 nM Bcd_FN_, 50 μM crotonyl-CoA, 100 μM NADH, 0.5 mg/ml of Ldh_RB_ and variable concentrations of 0.2 to 10 μM D-lactate. The reaction was initiated by the addition of 40 nM Ldh_FN_, and the absorbance change was monitored at 340 nm at 20 °C using a Lambda 265 spectrophotometer (PerkinElmer) housed in the glovebox. An absorbance decrease at 340 nm (indicating NADH oxidation) was observed when all reaction mixture components were present. For the reverse reaction (pyruvate + butyryl-CoA → lactate + crotonyl-CoA), a 1 ml reaction was prepared containing 40 nM ETF_FN_, 40 nM Bcd_FN_, 200 μM butyryl-CoA, 100 μM NAD^+^, 0.5 mg/ml of Ldh_LL_, and 0.5 mM pyruvate. The reaction was initiated with the addition of 40 nM Ldh_FN_, and the absorbance change was monitored at 340 nm at 20 °C.

### Redox titrations

As for the stopped-flow studies, redox titrations are performed under anaerobic conditions in the glovebox with an O_2_ < 5 ppm. Titrations were performed in 50 mM potassium phosphate buffer, pH 7.0, which was made anaerobic by extensive purging with N_2_ at 25 °C, followed >16 h of equilibration in the glovebox. The flavoproteins (ETF_FN_, Bcd_FN_, and Ldh_FN_) in 1 ml aliquots were introduced into the glovebox and equilibrated with the N_2_ atmosphere for >16 h at 4 °C. The proteins were diluted to ∼50 μM in a total volume of 7 ml in 50 mM potassium phosphate buffer, pH 7.0, with 10% (v/v) glycerol. Redox mediators)—benzyl viologen (*E*^o^′ = −359 mV), 2-hydroxy-1,4-napthoquinone (*E*^o^′ = −0.425 mV), and phenazine methosulfate (*E*^o^′ = 80 mV)—were added to the protein sample to a final concentration of 1 μM. The flavoproteins were gradually reduced with the addition of 1 μl aliquots of sodium dithionite. After each addition of dithionite and equilibration with the protein-redox mediator solution, the potential and the flavin absorbance spectrum were recorded. The potential was measured using a pH/ORP meter (Orion 3-Star Benchtop Meter, Thermo Fisher Scientific) equipped with an ORP electrode (Orion 9179 BNMD, Thermo Fisher Scientific), and the flavin absorbance spectra were recorded with a Lambda 265 UV/visible spectrophotometer positioned in the glovebox. The observed potentials obtained with the Ag/AgCl electrode were normalized to the normal hydrogen electrode by adding 204 mV to the potential values.

### Spectral deconvolution

The spectral and redox profiles of ETF_FN_ and Ldh_FN_ were determined by MCR-ALS analysis of the entire spectral dataset using MATLAB (Mathworks) for Windows. MCR intends to recover the pure response profiles (spectra, redox profile) of a mixture without any underlying assumptions or known information. The ALS is the method used to solve the MCR model, implemented in the MCR-ALS GUI 2.0 (https://mcrals.wordpress.com/download/). The analysis was constrained with no-negative absorbance spectra for all components. The number of components was determined by singular value decomposition. The midpoint potential for the oxidized-anionic semiquinone (FAD/FAD^•-^) transition and anionic semiquinone-hydroquinone transition (FAD^•-^/FADH^-^) were determined from the extracted redox profiles, where the relative concentrations of the components of the redox couple are equal.

## Data availability

All data are contained within the article and supporting information.

## Supporting information

This article contains supporting information (21, 40, 77, 78).

## Accession codes

Ldh ((S)-2-hydroxy-acid oxidase) from *F. nucleatum* subsp. *polymorphum* ATCC 10953 Genebank accession: AAL93662.1; Bcd from *F. nucleatum* subsp. *polymorphum* ATCC 10953 Genebank accession: ZP_02272040; EtfB from *F. nucleatum* subsp. *polymorphum* ATCC 10953 Genebank accession: ZP_02272039; and EtfA from *F. nucleatum* subsp. *polymorphum* ATCC 10953 Genebank accession: ZP_02272038.

## Conflict of interest

The authors declare that they have no conflicts of interest with the contents of this article.
