# Supplementary material for: Direct coupling of lactate oxidation with butyryl-CoA formation via a canonical electron transfer flavoprotein in Fusobacterium nucleatum
Source: J Biol Chem. 2025 Oct 9;301(11):110796. doi: 10.1016/j.jbc.2025.110796 (PMC12630350; doi:10.1016/j.jbc.2025.110796)
Supplement: Supporting Tables and Figures [file mmc1.docx]

**Supporting Information**

**Direct coupling of lactate oxidation with butyryl-CoA formation via an electron transfer protein in *Fusobacterium nucleatum***

Long T.M. Do, Robert Godin and Kirsten R. Wolthers^1^*

^1^Department of Chemistry, University of British Columbia, Okanagan campus, 3247 University Way, Kelowna, Canada

Keywords: *Fusobacterium nucleatum*, electron transfer flavoprotein, butyryl-CoA dehydrogenase, LrgAB, D-lactate dehydrogenase

Corresponding Author: Kirsten Wolthers

kirsten.wolthers@ubc.ca

**Table S1**. List of oligonucleotides used for cloning Ldh, Bcd, and Etf. The restriction site present in the oligonucleotide is shown in bold type. The sequences are written 5ʹ to 3ʹ.

| Name of Primers | Sequences |
| --- | --- |
| Forward LDH | ATACATC**CATATG**GGAAGTCATGTGTACAACAAAGTAAGTGAAG |
| Reverse LDH | TGA**GGATCC** TTA TGC CTT ATA ACA AAC TTT ATT TGG ATT TAA AAT C |
| Forward BCD | TGA **GGA TCC** ATG GCA TAT TTA ATT TCT GAA GAA GCT TCA AG |
| Reverse BCD | CG **GAA TTC** CTA ACG CCC AAT TAC ATT ATT AGC AAT TAC AAT TC |
| **Forward ETF** | ATACATC**CATATG**GAAATATTAGTTTGTATAAAACAAGTTGCAGATGAC |
| **Reverse ETF** | TGA **GGATCC** CTA AGA TTT TAT TTT TTT AAT TTC CTC TAT CAT AAG TGG |

A woodii --MSKILVCIKQVPGT-SNVEVDPETGVLIRDGVESKLNPYDLFGLETAFRLKEQLG-G- 55

A fermentans ---MNIVVCVKQVPDT-AEMKIDPVTNNLVRDGVTNIMNPYDQYALETALQLKDELG-A- 54

FN0784 ---MRIVVCIKQVPDT-TEVKIDPVKGTIIRDGVPSIMNPDDKGGLEEALKLKDLYG-A- 54

C difficile ---MNIVVCIKQVPDT-TEVKLDPNTGTLIRDGVPSIINPDDKAGLEEAIKLKEEMG-A- 54

FN1534 ---MEILVCIKQVADDSVEVFMNEKTGKPALEGVEKVVNAFDTYALEMAVRLKETKG-DI 56

M methylotrophus ---MKILVAVKQTAALEEDFEIREDGMDVDEDFMMYDLNEWDDFSLEEAMKIKESSDTDV 57

H sapiens MAELRVLVAVKRVIDYAVKIRVKPDRTGVVTDGVKHSMNPFCEIAVEEAVRLKEKKL-VK 59

P denitrifcans ---MKVLVPVKRLIDYNVKARVKSDGSGVDLANVKMSMNPFDEIAVEEAIRLKEKGQ-AE 56

A. woodii TITTLSMGPMQSKEVLMESFYMGADEGCLLS--DRKFGGADVVATSYTLAQGTKRLG--- 110

A fermentans HVTVITMGPPHAESVLRDCLAVGADEAKLVS--DRAFGGADTLATSAAMANTIKHFG--- 109

FN0784 EVIVITMGPPQAEAILREAYAMGADRAILIT--DRKFGGADTLATSNTIAAAIRKIE--- 109

C difficile HVTVITMGPPQADMALKEALAMGADRGILLT--DRAFAGADTWATSSALAGALKNI---- 108

FN1534 TVITLSLGGEDAKNGLKNCLAVGADEAFHIK--DENYQEKDAVIIAQALFKGIQKIEEQR 114

M methylotrophus EVVVVSVGPDRVDESLRKCLAKGADRAVRVW--DDAAEGSDAIVVGRILTEVIKKE---- 111

H sapiens EVIAVSCGPAQCQETIRTALAMGADRGIHVEVPPAEAERLGPLQVARVLAKLAEKE---- 115

P denitrifcans EIIAVSIGVKQAAETLRTALAMGADRAILVVAADDVQQDIEPLAVAKILAAVARAE---- 112

A woodii --DFDLIICGKQTTD**G**DTAQVGPEMAEFLGIPHVTNVIKILAAD-EKGLTLQMNMEES-L 166

A fermentans --VPDLILCGRQAID**G**DTAQVGPEIAEHLGLPQVTAALKVQVK--DDTVVVDRDNEQM-S 164

FN0784 --DIDLIVAGRQAID**G**DTAQVGPQIAEHLGLPQVSYVKEMEYKEDSKSFVIKRATEDG-Y 166

C difficile --DFDIIIAGRQAID**G**DTAQVGPQIAEHLNLPSITYAEEIKT--EGEYVLVKRQFEDC-C 163

FN1534 GKKFDIIFCGKETTD**F**AAGQVGIMLADELNYGVVTNLVDIDT--EGEKVIAKKETETG-Y 171

M methylotrophus --APDMVFAGVQSSD**Q**AYASTGISVASYLNWPHAAVVADLQYKPGDNKAVIRRELEGGML 169

H sapiens --KVDLVLLGKQAID**D**DCNQTGQMTAGFLDWPQGTFASQVTLE--GDKLKVEREIDGG-L 170

P denitrifcans --GTELIIAGKQAID**N**DMNATGQMLAAILGWAQATFASKVEIE--GAKAKVTREVDGG-L 167

A woodii EIQRVPYPCLITVDKDIYTPRLPSYKRKLDISKNPEIKILTLKDMYDTNEKKYGLSGSPT 226

A fermentans MTFTMKMPCVVTVMRSKDL-RFASIRGKMKA-RKAEIPVYTAAAL-EIPLDIIGKAGSPT 221

FN0784 FLLELPTPGLVTVLSEANQPRYMNVGAIVDVFERP-IETWTFDDI-EIDPAKIGLAGSPT 224

C difficile HDLKVKMPCLITTLKDMNTPRYMKVGRIYDAFENDVVETWTVKDI-EVDPSNLGLKGSPT 222

FN1534 EKVEVASPCLVTVNKPNYEPRYPTIKSKMAARKKEIAEVSTEVA-----------NESAV 220

M methylotrophus QEVEINCPAVLTIQLGINKPRYASLRGIKQAATKPIE-EVSLADI-GLSANDVGAAQSMS 227

H sapiens ETLRLKLPAVVTADLRLNEPRYATLPNIMKAKKKKIE-VIKPGDL-GVDLTSKLSVIS-- 226

P denitrifcans QTIAVSLPAVVTADLRLNEPRYASLPNIMKAKKKPLD-EKTAADY-GVDVAPRLEVVS-- 223

A woodii QVERIFPPESNVE-KTSFE-GDGKVLAKALLGILTEKKYLG--- 265

A fermentans QVMKSFTPKVTQVHGEIFDDEDPAVAVDKLVNKLIEDKIITK-- 263

FN0784 KVNKSFTKGVKEP-GVLHE-VDAKEAANIILEKLKEKFII---- 262

C difficile SVFKSFTKSVKPA-GTIYN-EDAKTSAGIIIDKLKEKYII---- 260

FN1534 KEVKLFSPPKRQA-GVKIKTGTAEELVAQAIQKMLEAKVF---- 259

M methylotrophus RVRRMYIPEKGRA-TMIEG--TISEQAAK----IIQIINEFKGA 264

H sapiens ----VEDPPQRTA-GVKVE--TTEDLVAK----LKEIGRI---- 255

P denitrifcans ----VREPEGRKA-GIKVG--SVDELVGK----LKEAGVI---- 252

### Figure S1. CLUSTAL 2.1 multiple sequence alignment of the β-subunits of Etfs. The conserved glycine residue in bifurcating ETFS is in bold, along with the bulkier side chain in canonical ETFs. The accession codes are as follows: *Methylophilus methylotrophus* (*Methylophilus* *methylotrophus*; PDB entry 1O96), *Homo sapiens* (*H. sapiens,* PDB entry: 1EFV), *P.* *denitrificans* (*Paracoccus denitrificans,* PDB entry: 1EFP); *A. woodii* (*Acetobacterium woodii*; PDB entry 6FAH); *Acidaminococcus fermentans* (*A. fermentans* PDB entry: 4KPU) and *C. difficile* (*Clostridium difficile*, PDB entry: 5OL2).

4

5

1

2

3

**
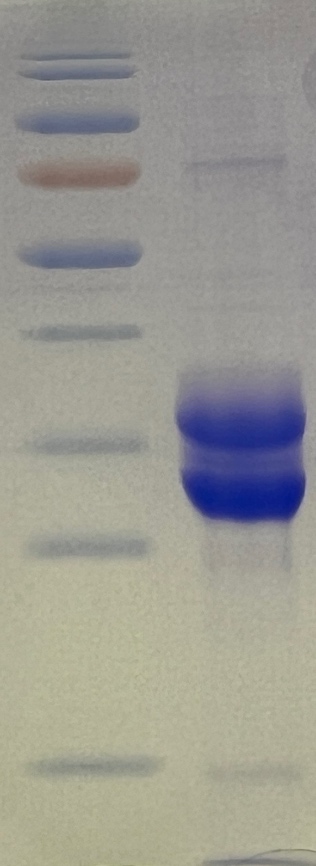

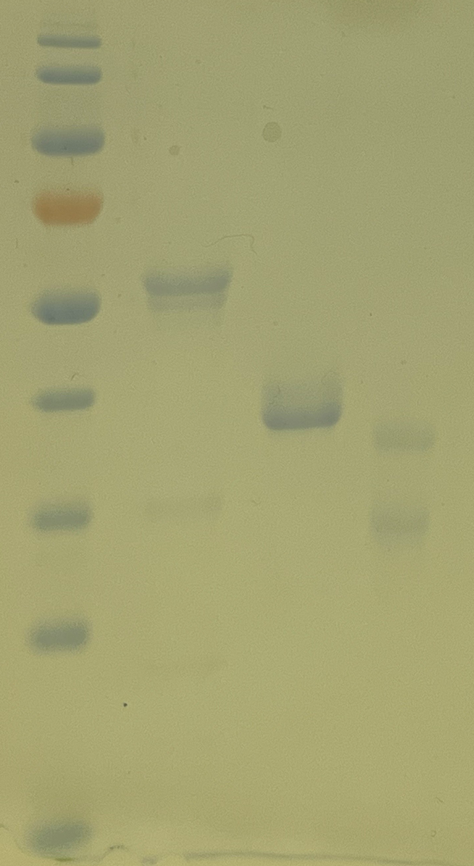
**

180

180

130

130

100

70

100

70

55

55

40

40

35

25

35

25

15

15

**Figure S2**.  **SDS 12% polyacrylamide gels of Ldh_FN_, Bcd_FN_ and ETF_FN_.** Left gel. Lane 1, PageRuler prestained protein ladder (MW from top to bottom of 180, 130, 100, 70, 55, 40, 35, 25, and 15 kDa); Lane 2, purified Ldh_FN_; Lane 3, purified Bcd_FN_; Right gel. Lane 4, same as lane 1; Lane 5, the α and β subunits of ETF_FN_.


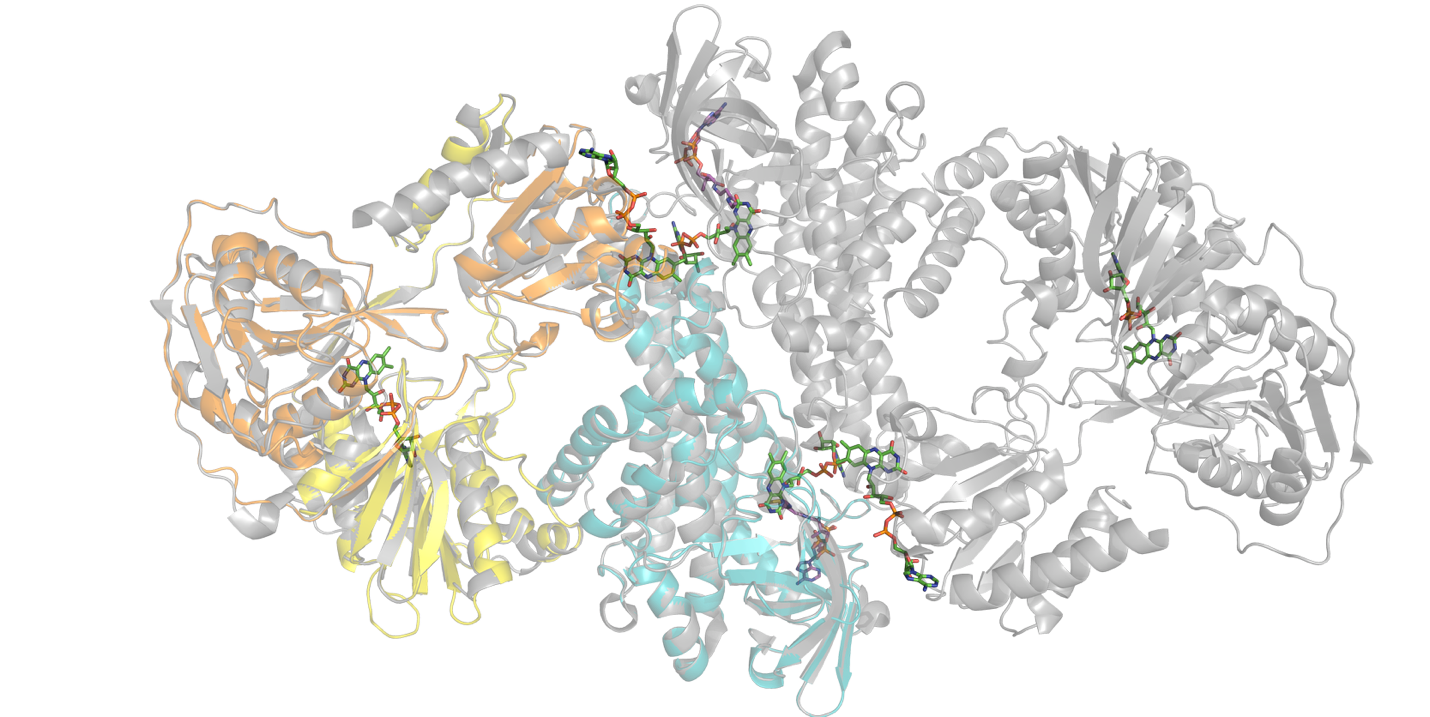
**Figure S3**. Structure of the Bcd-ETF complex from *C. difficile* (Bcd-ETF)_CD_ superimposed on a model of the (Bcd-ETF)_FN_ complex (PDB 5OL2). (Bcd-ETF)_CD_ forms a heterododecameric complex with four subunits of Bcd forming the core and four ETF units on the periphery (1). For clarity, only two subunits of Bcd_CD_ and ETF are shown. The models of Bcd_FN_, EtfA_FN_ and EtfB_FN_ are coloured cyan, orange and yellow, respectively. In Bcd-ETF_CD_, each of the three subunits, Bcd, EtfA and EtfB binds one FAD (isoalloxazine in stick format with the carbon atoms coloured green), but in ETF_FN_, the β-subunit binds AMP. The acyl-CoA derivative is shown in stick format with the carbons coloured purple.


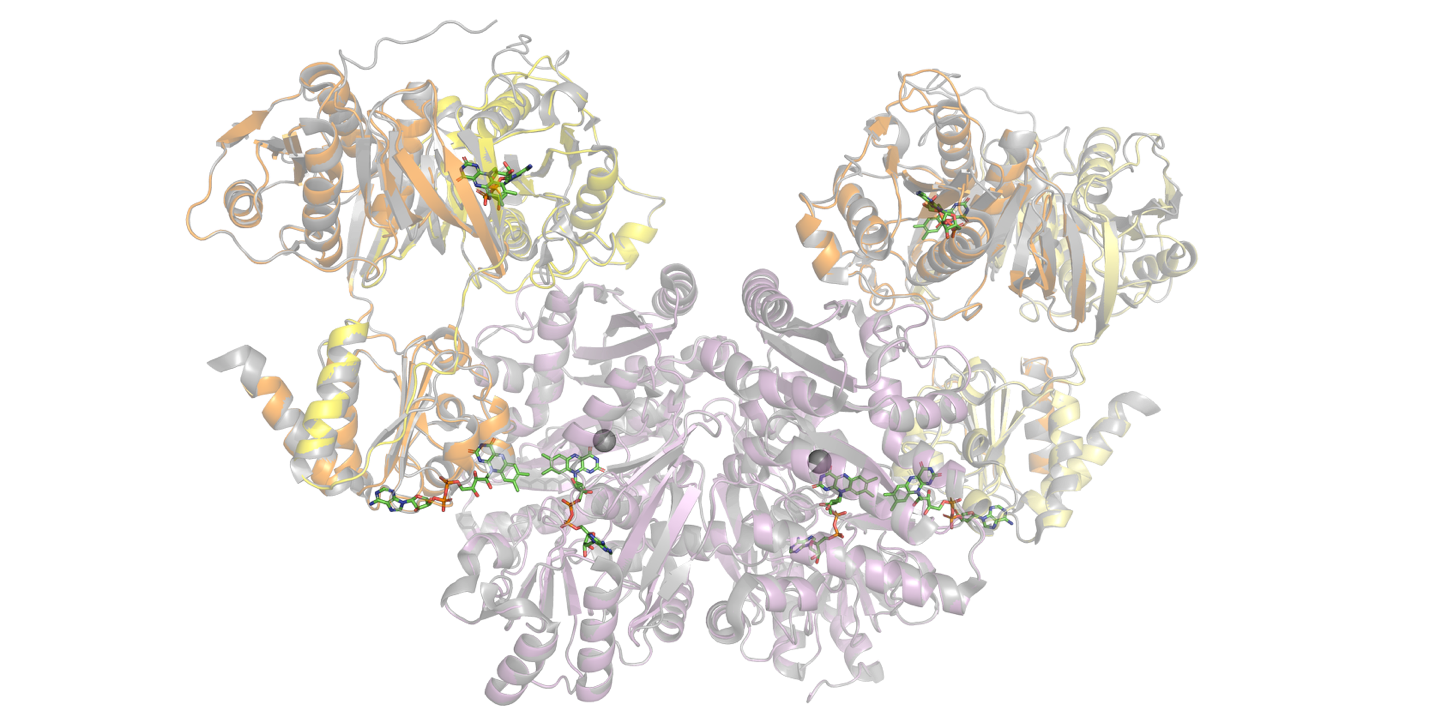


**Figure S4**. Cryo-EM structure of Ldh-ETF complex from *A. woodii* (PDB 7QH2) (2) superimposed on a model of the (Ldh-ETF)_FN_ complex generated by SWISS-MODEL(3). The (Ldh-ETF)_AW_ complex (shown in grey) is composed of an Ldh dimer, which forms the core and two ETF modules on the periphery, similar to that described for (Bcd-ETF)_CD._ The models of Ldh_FN_, EtfA_FN_ and EtfB_FN_ are coloured violet, orange and yellow, respectively. The FAD non-covalently associated to Ldh_AW_, EtfA_AW_ and EtfB_AW_ is shown in stick format with the carbon atoms coloured green.


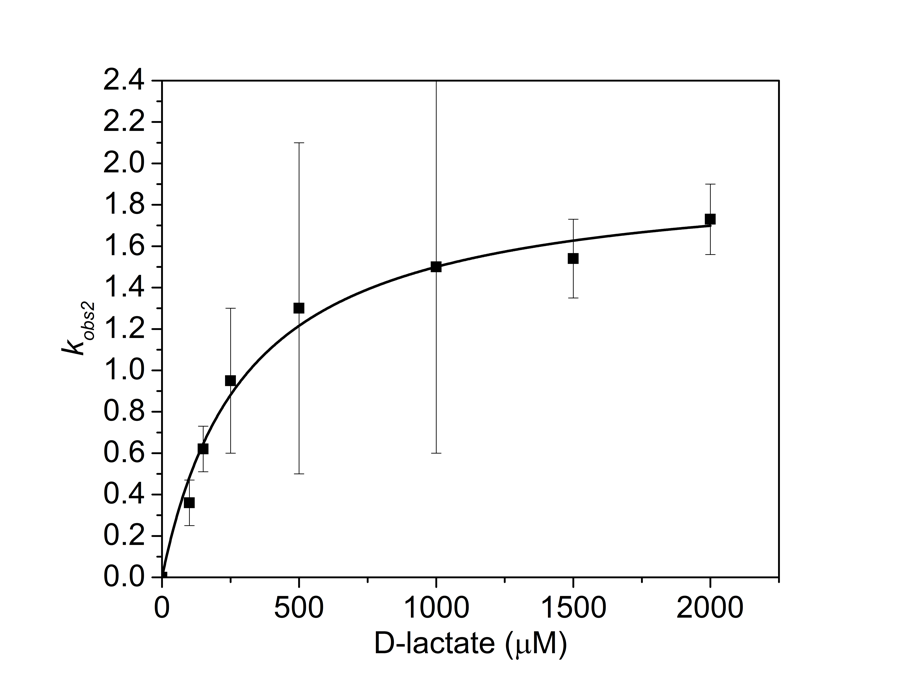


**Figure S5**. **Pre-state kinetic data associated with D-lactate reduction of Ldh_FN_** A plot of *k*_2obs_ versus the concentration of D-lactate. The data were fitted to a hyperbolic function to extract a *K*_d_ of 305 ± 44 μM and a limiting rate constant of reduction (*k*_lim_) of 2.1 ± 0.3 s^-1^.


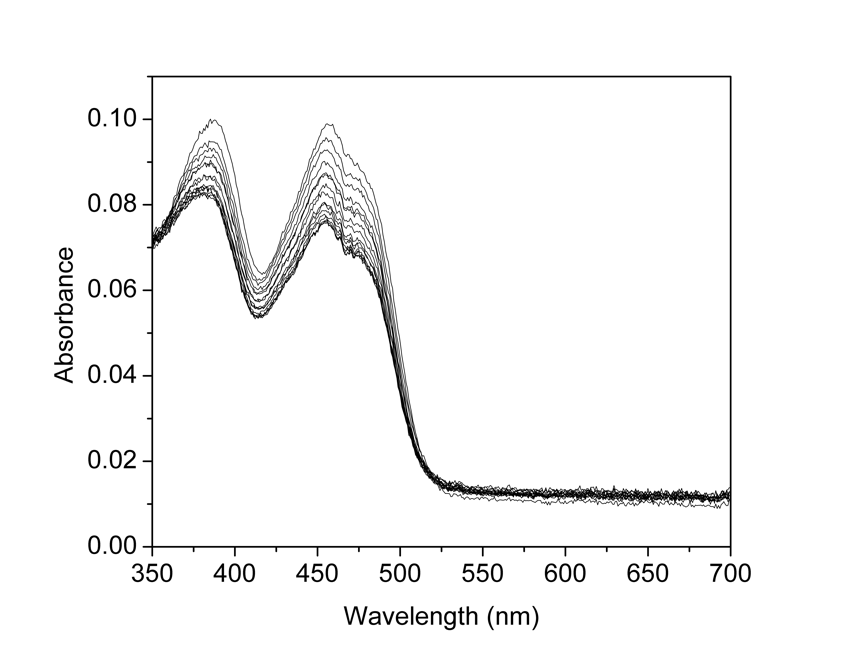

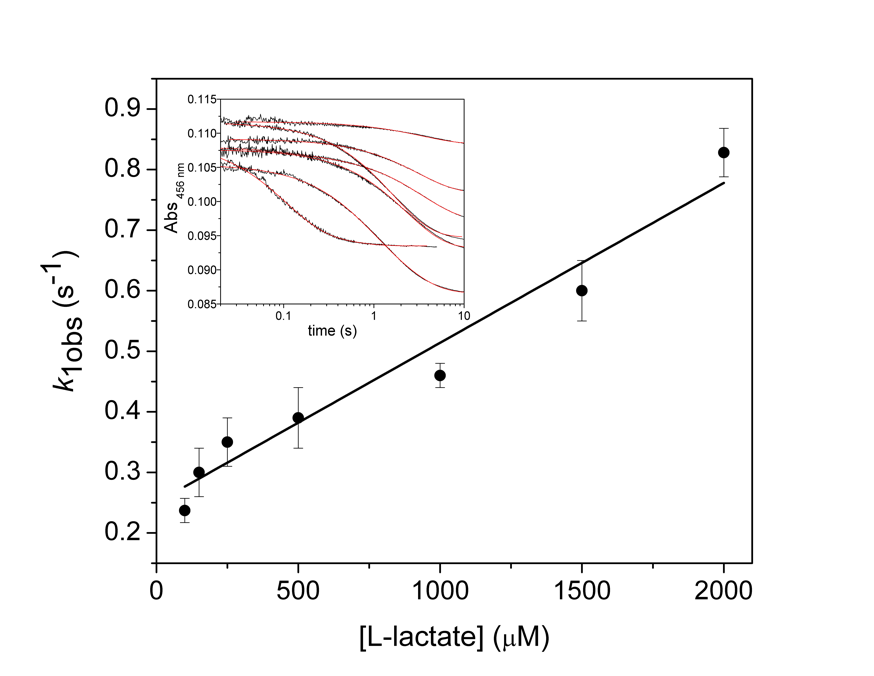


B

A

**Figure S6**. **Pre-state kinetic data associated with L-lactate reduction of Ldh**. (A) Multiwavelength absorbance changes following the rapid mixing of 1 mM L-lactate and 20 μM of Ldh_FN_ in 50 mM potassium phosphate buffer, pH 7.5 at 20 °C under anaerobic conditions as described in Methods and Materials. Spectra were collected over 30 s. (B) A plot of *k*_1obs_ versus the concentration of L-lactate. A linear fit of the data generated a *k*_on_ value of 2.8 × 10^-4^ μM^-1^ s^-1^. Inset: Single wavelength absorbance traces were collected at 456 nm at varying D-lactate concentrations and fitted to a single exponential equation to extract *k*_1obs_.


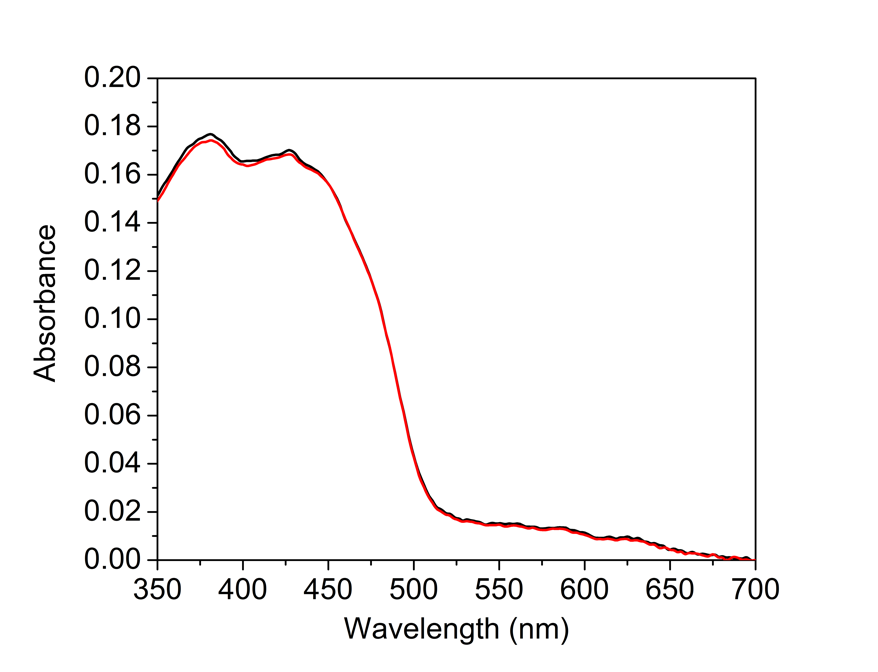


**Figure S7**. **Spectra of Bcd_FN_ with and without the addition of 200 μM butyryl-CoA.** UV-visible absorbance spectra of Bcd_FN_ before (black line) and after (red line) the addition of 200 μM butyryl-CoA. Spectra were collected under anaerobic conditions in a glovebox.

**A**

1UKW YVREFPVEKLLRDVKLNQI**Y**EGTNEIQRLIIARHILAA--- 379

3MDD FNTEYPVEKLMRDAKIYQI**Y**EGTAQIQRIIIAREHIGRYK- 385

1EGE FNTEYPVEKLMRDAKIYQI**Y**EGTSQIQRLIVAREHIDKYKN 396

BCDFN YSREYPVEKLIRDSKIFQI**F**EGTNEIQRIVIANNVIGR--- 378

1JQI YVTEMPAERYYRDARITEI**Y**EGTSEIQRLVIAGHLLRSYRS 388

5LNX YMKDYPVERLLRDAKVTQI**Y**EGTNEIQRLIISKYLLGGT-- 379

5OL2 YTRDYPVERMMRDAKITEI**Y**EGTSEVQRMVISGKLLK---- 378

4L1F YTVDYPAERYMRNAKITQI**Y**EGTNQVMRIVTSRALLRDKKK 383

1BUC YSEEYPVARHMRDAKITQI**Y**EGTNEVQLMVTGGALLR---- 383

**B**


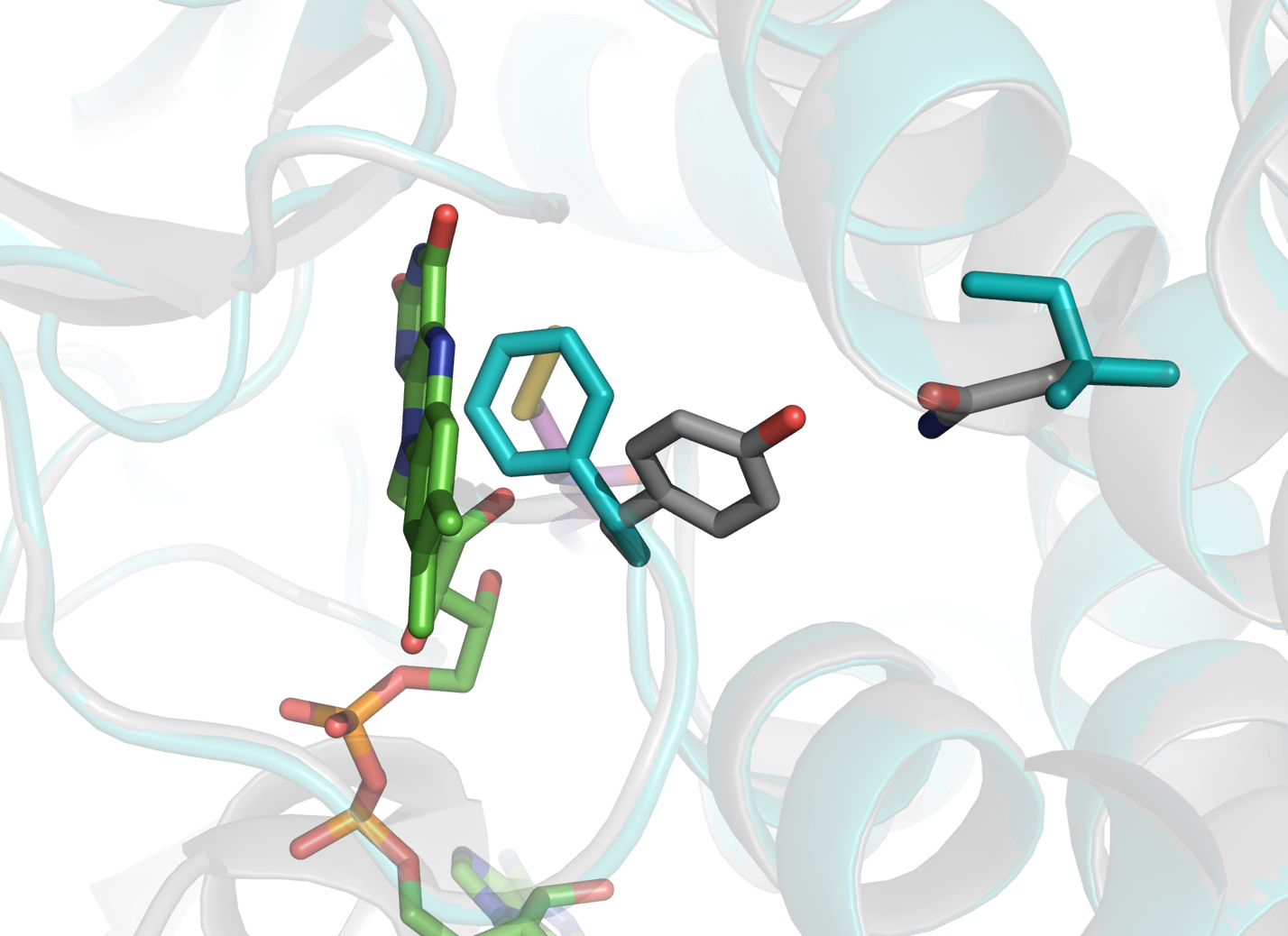


**Ile247**

**Tyr248**

**Phe360**

**Tyr361**

**Figure S8**. Comparison of Bcd active site. (A) CLUSTAL 2.1 multiple sequence alignment of the Bcd_FN_ with structurally determined Bcd homologs. The conserved tyrosine flanking the δ-FAD is in bold type. The PDB ID codes correspond to the following: 5OL2, acyl-CoA dehydrogenase from *Clostridioides difficile*; 4L1F, acyl-CoA dehydrogenase from *Acidaminococcus fermentans* DSM 20731; 1UKW, acyl-CoA dehydrogenase from *Thermus thermophilus*; 5LNX, acyl-CoA dehydrogenase from *Bacillus subtilis subsp. subtilis* str. 168; 1BUC, butyryl-CoA dehydrogenase from *Megasphaera elsdenii*; 3MDD, medium chain acyl-CoA dehydrogenase from *Sus scrofa*, 1JQI, short chain acyl-CoA dehydrogenase from *Rattus norvegicus*, 1EGE, medium chain acyl-CoA dehydrogenase from *Homo sapiens*. Bcd_FN_ is labelled BCDFN. (B) Structure of the Bcd-ETF complex from *C. difficile* (Bcd-ETF)_CD_ (PDB 5OL2) superimposed on a model of the (Bcd-ETF)_FN_ complex generated through SwissModel. The colouring scheme is the same as that described for Figure S3. Inspection of the substrate-binding cleft reveals that the conserved tyrosine (Tyr361) is replaced by Phe360 in Bcd_FN_

**
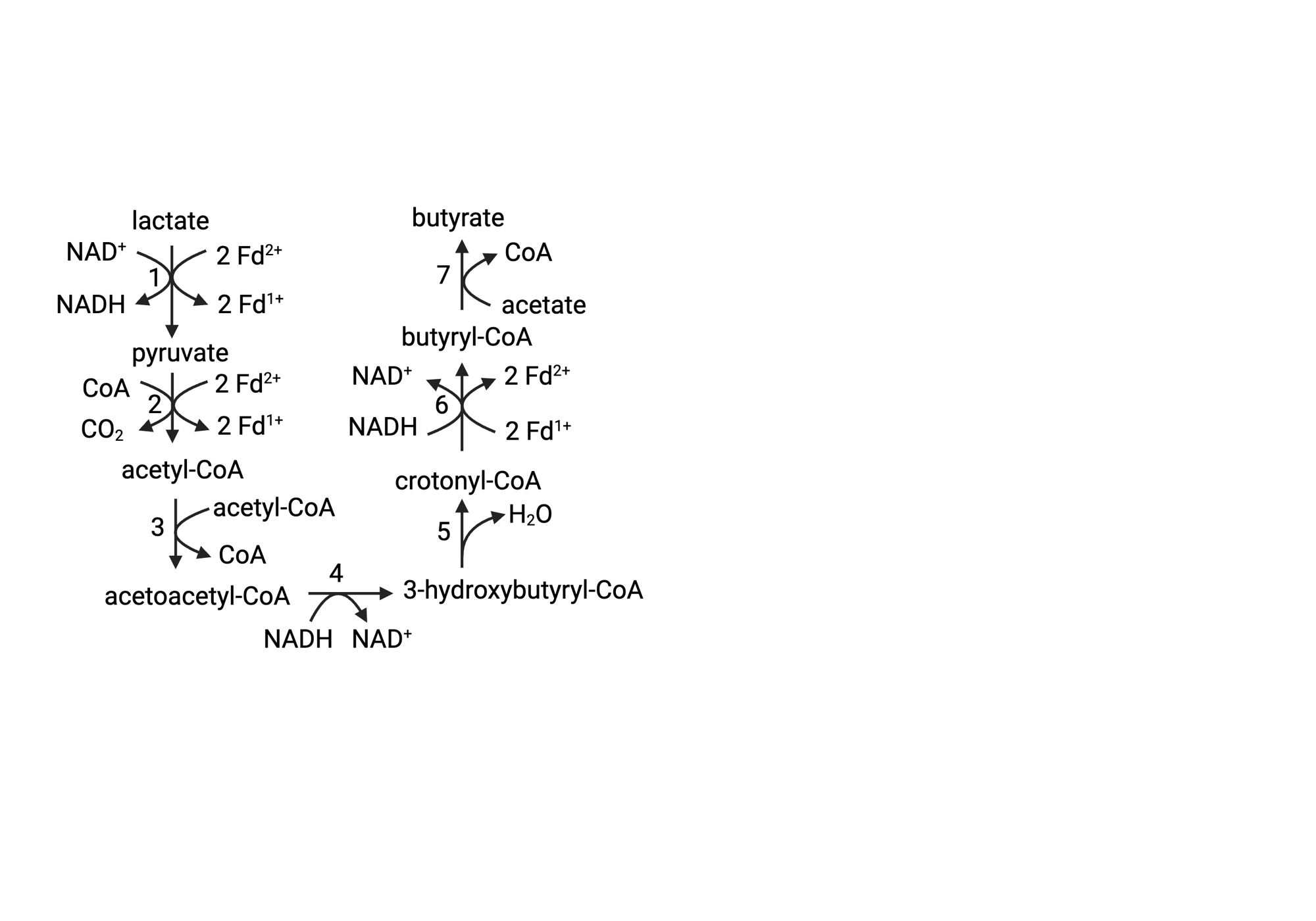
**

**Figure S9. Proposed scheme for butyrate conversion to lactate.** Lactate is oxidized by a confurcating Ldh-ETF complex (1) to form pyruvate which is converted to acetyl-CoA and CO2 by pyruvate ferredoxin oxidoreductase (2). Two equivalents of acetyl-CoA form acetoacetyl-CoA through an acetyl-CoA transferase (3). The acetoacetyl-CoA is reduced to 3-hydroxybutyryl-CoA by 3-hydroxybutyryl-CoA dehydrogenase (4) which undergoes condensation to crotonyl-CoA by 3-hydroxybutyryl-CoA dehydratase (5). Crotonyl-CoA is reduced to butyryl-CoA by a bifurcating Bcd-ETF complex

A. hallii MNICVCIKQVPDT-NEIKVDPVTHTLVRKGVPSIVNPFDTYAQEVGVRLKEKLGGK-VVV 58

C. beijerinckii MQILLCVKQVPDDSIEIHLDNEKKKPKLNGVSLVANAFDTYALELAVRFTEAHGGK-VSV 59

F. necrophorum MEILVCAKQVADDSVEIMLNPETGKPALEGVTEVVNAFDTYALEMATRLKEEKGGT-VCV 59

FN1534 MEILVCIKQVADDSVEVFMNEKTGKPALEGVEKVVNAFDTYALEMAVRLKETKGDITVIT 60

C. catus MEILVLIKQVPDDSVEIKLDPATGAPKLDGVEAVVNAFDTYALEMAKRYIEDNGGN-VTV 59

A. hadrus MNILVCIKQVPDDSVEIHLN-AEGTPDLANVTPVVNAFDTYALEMAARLKESSEGE-VTV 58

A. caccae MEILVCVKQVPDDSVEVHLT-AEGKPDLANITPVVNAFDTYALEMAARLKEAGEGE-ITV 58

1. hallii ISMGPEQAKEAIKTCLSVGADAGYLISSRKFGGSDTLATSYILSEAIKAVEEKEGLKFDL 118
2. beijerinckii LTVGADDSLNTLKNCLAVGAKEAFFVKDDLYADLDAMGTADVLADAIHKIEADKGEKFDL 119

F. necrophorum VSLGGESAANSLKNCLAVGADEAFHIKDEEYQNRDTLAVAQKVAEGIQKIEEQRGKKFDV 119

FN1534 LSLGGEDAKNGLKNCLAVGADEAFHIKDENYQEKDAVIIAQALFKGIQKIEEQRGKKFDI 120

C. catus ATIGDDDATAALRTCLAVGAGKAFLIKDASFAGSDTTAKAYILSKAVAKIEEINGAKFDI 119

A. hadrus VCVGEDSAKNSLKNCLAVGADHAFLVSDDAFKGSDTTGIANILKNVIAKLEADNGQKFDL 118

A. caccae LCIGGEEARNSLKNCLAVGADHAYLVRDDRFDGSDSRGISHILMCAAKKIEEDTGKKFDL 118

1. hallii ILCGKQAVD**G**DTAQVGPEIAEHLGLPQITYALDIIEKDGDIQVKRECDEGYDMISTQLPA 178

C. beijerinckii ILCGKESTD**E**ITGQVGAMLAEKLKTGYVSSAIEIDLKDNSMEIHQETEEGYNLVSLECPA 179

F. necrophorum IFCGRESTD**Y**ASSQVGIMIADGLGYGVVSNLVDIEADETKVIAKRETEEGYQRIEVAVPC 179

FN1534 IFCGKETTD**F**AAGQVGIMLADELNYGVVTNLVDIDTEGEKVIAKKETETGYEKVEVASPC 180

C. catus VFCGKEATD**F**SKGMVGVQLASELGVGVATDVIAVDPAEGGVTVKQETETGYNVIEMTTPC 179

A. hadrus VFCGKEATD**A**ALGQGGLMLAEELGTSVITNITEIALDGDKVTAKQETEEGYRTVEASAPC 178

A. caccae IFCGKEATD**A**ALGQVGAELSELLGTGVITNVIGVEFEEGQVCAKQETEEGYRLIETASPC 178

A. hallii VVTVVRLPYEPRYPTIKSKMAAKKKEIPVLTEEDIPAISLERCGLSGSPTKVKKTYTPVT 238

C. beijerinckii VVTVSKPDYDPRYPTIKSKMASRKAVIPTYSAAEIGEVK-------QAKVRCIEYVAPPK 232

F. necrophorum VVTVNKPNYEPRYPTIKSKMAARKKAIAEVVVDS----------KAENIVKEVAISAPPK 229

FN1534 LVTVNKPNYEPRYPTIKSKMAARKKEIAEVSTEV----------ANESAVKEVKLFSPPK 230

C. catus VVTIQKPDYDPRYPTIKTKMAARKATINEITAADLA-VDAAKIGEAGSLTKVLKLYEPPK 238

A. hadrus VVTVTKPEYDPRYPTIKNKMAARKKPIGDIKEADLADLEKEKVGESNAKVQIVKLYEPAK 238

A. caccae VLTVTKPDYDPRYPTIKNKMAARKKPIGTIKAEDLSMLELEKTGEDGAFVKTLKLYEPPK 238

A. hallii EKNGVKLEGMEAEDAAKEVVKLIYDAKIL 267

C. beijerinckii KEAGIKIQEKDATLAVSAVMEQMKKDKAI 261

F. necrophorum RQAGIKIKSGNAEELVAQAIEKMLEAKVF 258

FN1534 RQAGVKIKTGTAEELVAQAIQKMLEAKVF 259

C. catus KQAGVKIQEETVADSTMRALAMIAEAKVL 267

A. hadrus KEAGIKIQEETPEDSAIKAVAMMADAKVF 267

A. caccae KEAGIKIQEETAQDSAFKAVAMMADAKVF 267

**Figure S10**. CLUSTAL 2.1 multiple sequence alignment of the β-subunits of ETFs. The conserved glycine residue present in bifurcating ETF is in bold along with the bulkier side chain present in canonical ETFs.


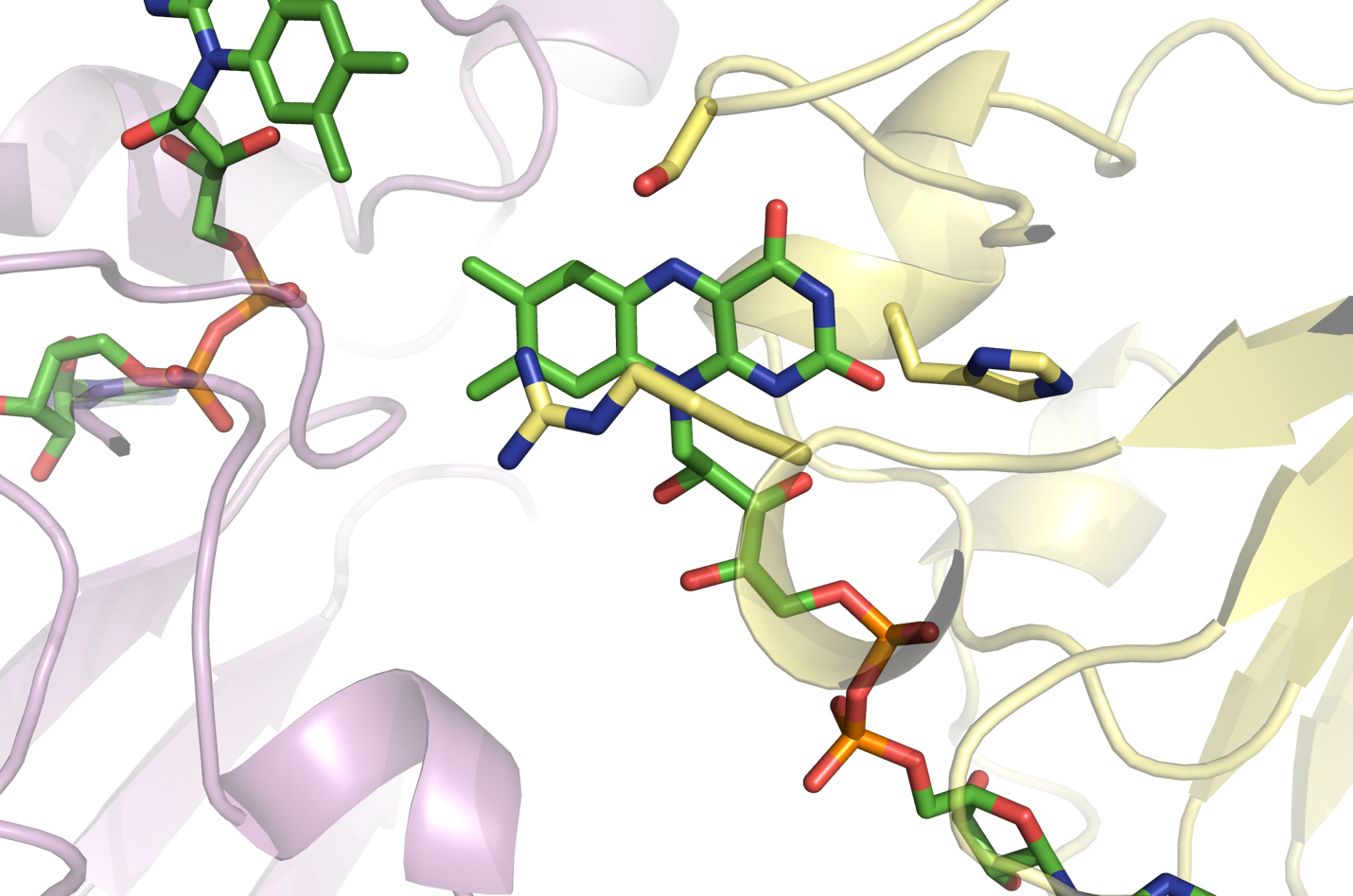


His274

Ser254

Arg237

**Figure S11.** Model of the α-FAD active site of ETF_FN_ showing the location of Arg237, His274 and Ser254.

1. Demmer, J. K., Pal Chowdhury, N., Selmer, T., Ermler, U., andBuckel, W. (2017) The semiquinone swing in the bifurcating electron transferring flavoprotein/butyryl-CoA dehydrogenase complex from Clostridium difficile Nat Commun **8**, 1577 10.1038/s41467-017-01746-3

2. Kayastha, K., Katsyv, A., Himmrich, C., Welsch, S., Schuller, J. M., Ermler, U., andMuller, V. (2022) Structure-based electron-confurcation mechanism of the Ldh-EtfAB complex Elife **11**, 10.7554/eLife.77095

3. Waterhouse, A., Bertoni, M., Bienert, S., Studer, G., Tauriello, G., Gumienny, R. *et al.* (2018) SWISS-MODEL: homology modelling of protein structures and complexes Nucleic Acids Res **46**, W296-W303 10.1093/nar/gky427

4. Chowdhury, N. P., Kahnt, J., andBuckel, W. (2015) Reduction of ferredoxin or oxygen by flavin-based electron bifurcation in Megasphaera elsdenii FEBS J **282**, 3149-3160 10.1111/febs.13308
